# Supplementary material for: Structural dynamics of melting and glass formation in a two-dimensional hybrid perovskite
Source: Nat Commun. 2025 Aug 18;16:7696. doi: 10.1038/s41467-025-61410-z (PMC12361452; doi:10.1038/s41467-025-61410-z)
Supplement: Supplementary file 1 — Supporting Information [file 41467_2025_61410_MOESM1_ESM.pdf]

Supplementary Information

Structural Dynamics of Melting and Glass Formation in a Two-dimensional Hybrid Perovskite

Chumei Ye[1,2], Lauren N. McHugh[3]*, Pierre Florian[4], Ruohan Yu[5], Celia Castillo-Blas[1], Celia Chen[1,2], Arad Lang[1], Yuhang Dai[6], Jingwei Hou[7,8], David A. Keen[9], Siân E. Dutton[2]*, Thomas D. Bennett[1,10]*

[1] Department of Materials Science and Metallurgy, University of Cambridge, Cambridge CB3 0FS, UK. E-mail: tdb35@cam.ac.uk.

[2] Cavendish Laboratory, University of Cambridge, Cambridge CB3 0HE, UK.
E-mail: sed33@cam.ac.uk.

[3] Department of Chemistry, University of Liverpool, Liverpool L69 7ZD, UK.
E-mail: L.N.Mchugh@liverpool.ac.uk.

[4] CNRS, CEMHTI UPR3079 University of Orléans, Orléans 45000, France.

[5] State Key Laboratory of Advanced Technology for Materials Synthesis and Processing, Wuhan University of Technology, Wuhan 430070, P. R. China.

[6] Department of Engineering Science, University of Oxford, Oxford OX1 3PJ, UK.

[7] School of Chemical Engineering, The University of Queensland, St Lucia, QLD 4072, Australia.

[8] ARC Centre of Excellence for Green Electrochemical Transformation of Carbon Dioxide, Brisbane 4072, Australia.

[9] ISIS Facility, Rutherford Appleton Laboratory, Harwell Campus, Didcot OX11 0QX, UK.

[10] MacDiarmid Institute for Advanced Materials and Nanotechnology, School of Physical and Chemical Sciences, University of Canterbury, Christchurch 8140, New Zealand.
E-mail: thomas.bennett@canterbury.ac.nz.

* Corresponding authors

Contents

Supplementary Figures

Supplementary Fig. 1. Optical images of as-synthesised, glassy and recrystallised samples. 4
Supplementary Fig. 2. Pawley refinement of PXRD for crystalline (*S*-NEA)$_2$PbBr$_4$. 5
Supplementary Fig. 3. Simultaneous DSC-TGA experiment on (*S*-NEA)$_2$PbBr. 6
Supplementary Fig. 4. PXRD pattern of as-synthesised, glassy and recrystallised samples. 7
Supplementary Fig. 5. Pawley refinement of PXRD for recrystallised (*S*-NEA)$_2$PbBr$_4$. 8
Supplementary Fig. 6. Fragility of the glass-forming liquid (*S*-NEA)$_2$PbBr$_4$. 9
Supplementary Fig. 7. Pawley refinement of PXRD for crystalline (*R*-NEA)$_2$PbBr$_4$. 11
Supplementary Fig. 8. Pawley refinement of PXRD for crystalline (*rac*-NEA)$_2$PbBr$_4$. 12
Supplementary Fig. 9. Simultaneous DSC-TGA on (*R*-NEA)$_2$PbBr$_4$ and (*rac*-NEA)$_2$PbBr$_4$. 13
Supplementary Fig. 10. DSC experiment on (*R*-NEA)$_2$PbBr$_4$ in argon. 14
Supplementary Fig. 11. Scanning electron microscopy images of crystals and glasses. 15
Supplementary Fig. 12. SEM-EDS of (*S*-NEA)$_2$PbBr$_4$ and a_g(*S*-NEA)$_2$PbBr$_4$. 16
Supplementary Fig. 13. FT-IR spectra of (*S*-NEA)$_2$PbBr$_4$ and a_g(*S*-NEA)$_2$PbBr$_4$. 17
Supplementary Fig. 14. STEM images of (*S*-NEA)$_2$PbBr$_4$. 18
Supplementary Fig. 15. HAADF-STEM analysis of crystalline (*S*-NEA)$_2$PbBr$_4$. 19
Supplementary Fig. 16. STEM images of a_g(*S*-NEA)$_2$PbBr$_4$. 20
Supplementary Fig. 17. Ambient-temperature X-ray pair distribution functions. 21
Supplementary Fig. 18. Calculated total and partial X-ray pair distribution functions. 23
Supplementary Fig. 19. X-ray total scattering data for ball-milled glass a_m(*S*-NEA)$_2$PbBr$_4$. 24
Supplementary Fig. 20. DFT-calculated NMR spectra and atom assignments. 25
Supplementary Fig. 21. High-speed high-field ^{13}C and ^{1}H MAS NMR spectra. 26
Supplementary Fig. 22. High-speed high-field MAS NMR ^{1}H{^{1}H} DQ/SQ spectra. 27
Supplementary Fig. 23. Variable-temperature X-ray total scattering. 28
Supplementary Fig. 24. Variable-temperature X-ray pair distribution functions. 29
Supplementary Fig. 25. Gaussian peak fitting of VT-PDF data. 30
Supplementary Fig. 26. Individual peak analysis of VT-PDF data. 32
Supplementary Fig. 27. High-temperature X-ray total scattering. 33
Supplementary Fig. 28. High-temperature X-ray pair distribution functions. 34
Supplementary Fig. 29. Comparison of total scattering data from different beamlines. 35
Supplementary Fig. 30. Temperature-resolved *in situ* THz/Far-IR spectra. 36
Supplementary Fig. 31. *In situ* variable-temperature ^{1}H MAS NMR spectra for crystal. 37
Supplementary Fig. 32. *In situ* variable-temperature ^{13}C MAS NMR spectra for crystal. 38
Supplementary Fig. 33. *In situ* variable-temperature ^{207}Pb MAS NMR spectra for crystal. 39
Supplementary Fig. 34. High-temperature ^{13}C and ^{1}H MAS NMR spectra for liquid. 40
Supplementary Fig. 35. *In situ* variable-temperature ^{1}H MAS NMR spectra for glass. 41
Supplementary Fig. 36. High-temperature ^{1}H and ^{13}C MAS NMR spectra for glass. 42
Supplementary Fig. 37. High-temperature ^{207}Pb MAS NMR spectra for glass. 43
Supplementary Fig. 38. Ambient-temperature UV-vis spectra for crystal and glass. 44
Supplementary Fig. 39. Nanoindentation results for crystal and glass. 45

Supplementary Tables

Supplementary Table 1.
Crystallographic data from Pawley refinement of PXRD for crystalline (*S*-NEA)$_2$PbBr$_4$. 5
Supplementary Table 2.
Crystallographic data from Pawley refinement of PXRD for recrystallised (*S*-NEA)$_2$PbBr$_4$. 8
Supplementary Table 3.
Crystallographic data from Pawley refinement of PXRD for crystalline (*R*-NEA)$_2$PbBr$_4$. 11
Supplementary Table 4.
Crystallographic data from Pawley refinement of PXRD for crystalline (*rac*-NEA)$_2$PbBr$_4$. 12
Supplementary Table 5.
Elemental analysis of (*S*-NEA)$_2$PbBr$_4$ and a_g(*S*-NEA)$_2$PbBr$_4$. 16
Supplementary Table 6.
CHN microanalysis of (*S*-NEA)$_2$PbBr$_4$ and a_g(*S*-NEA)$_2$PbBr$_4$. 17
Supplementary Table 7.
Fitting parameters of the Gaussian fits for ambient-temperature PDF data. 22
Supplementary Table 8.
Fitting parameters of the Gaussian fits for variable-temperature PDF data. 31
Supplementary Table 9.
Thickness measurements of samples used in the UV-Vis experiment. 44
Supplementary Table 10.
Comparison of the mechanical properties of crystal and glass. 46

References 47

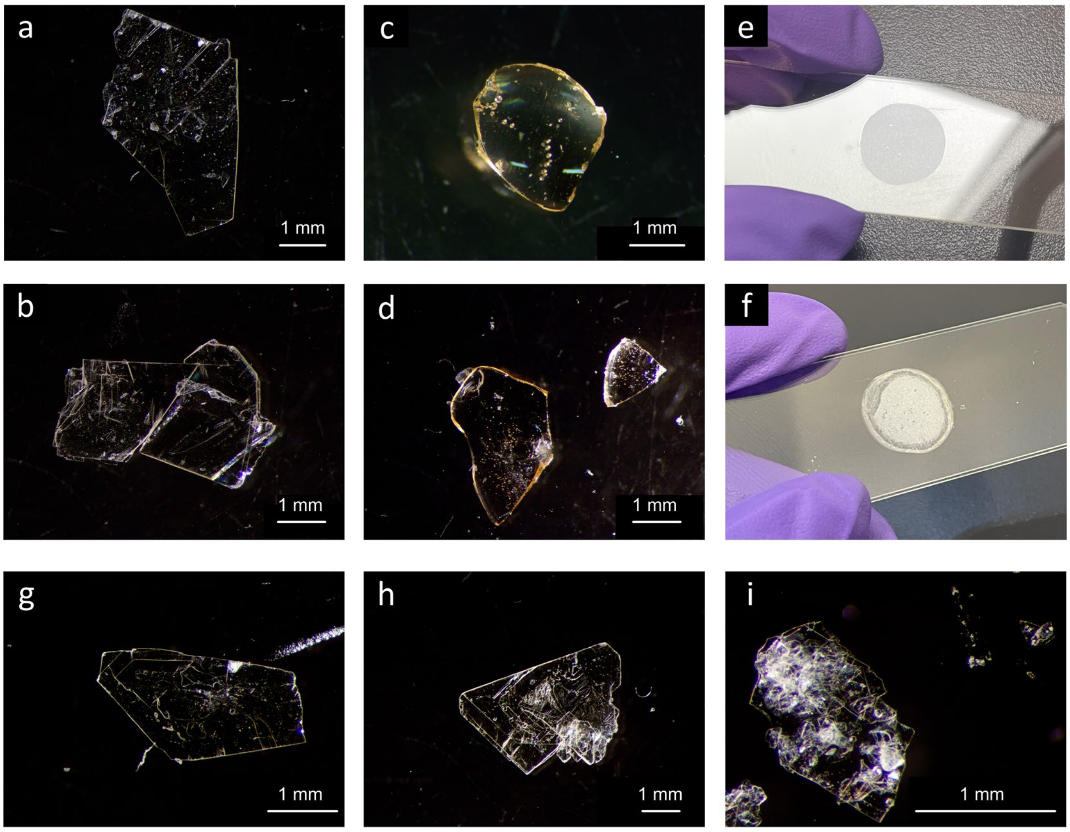

Supplementary Fig. 1. Optical microscope images of the as-synthesised, glassy and recrystallised samples. (a-b) As-synthesised (*S*-NEA)$_2$PbBr$_4$ crystals. (c-d) Bulk a_g(*S*-NEA)$_2$PbBr$_4$ glass formed in (c) argon and (d) air. (e) a_g(*S*-NEA)$_2$PbBr$_4$ film sandwiched between two glass slides and (f) the corresponding recrystallised film. (g-h) As-synthesised (*R*-NEA)$_2$PbBr$_4$ crystals. (i) As-synthesised (*rac*-NEA)$_2$PbBr$_4$ crystals. In (e) and (f), the average thicknesses of the film samples were measured using a micrometer screw gauge with a precision of 0.01 mm, yielding approximately 0.09 mm and 0.11 mm, respectively.

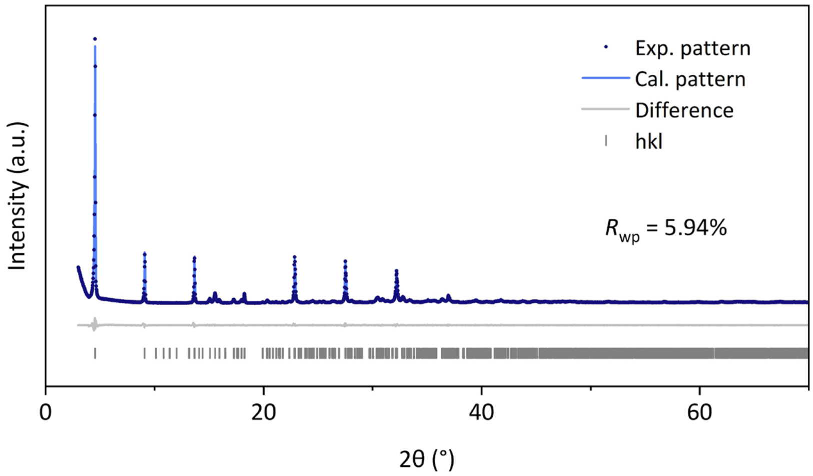

Supplementary Fig. 2. Pawley refinement of the PXRD for crystalline (S-NEA)$_2$PbBr$_4$, along with patterns simulated from the published Crystallographic Information File (CCDC 2015618)[1]. Experimental data (dark blue dots), calculated diffraction pattern (blue line), difference function (grey line) and symmetry-allowed reflections (grey ticks). X-ray wavelength = 1.5418 Å.

Supplementary Table 1.

Crystallographic data from Pawley refinement of the PXRD for crystalline (S-NEA)$_2$PbBr$_4$.

R_{wp}-value	Space Group	Lattice Parameters	Reported Lattice Parameters[1]
5.94%	$P2_1$	a = 8.7529 (14) Å<br>b =7.9635 (11) Å<br>c = 19.4994 (5) Å<br>α = 90°<br>β = 93.861 (13)°<br>γ = 90°	a = 8.75370 (19) Å<br>b = 7.95502 (16) Å<br>c = 19.5038 (5) Å<br>α = 90°<br>β = 93.806 (2)°<br>γ = 90°

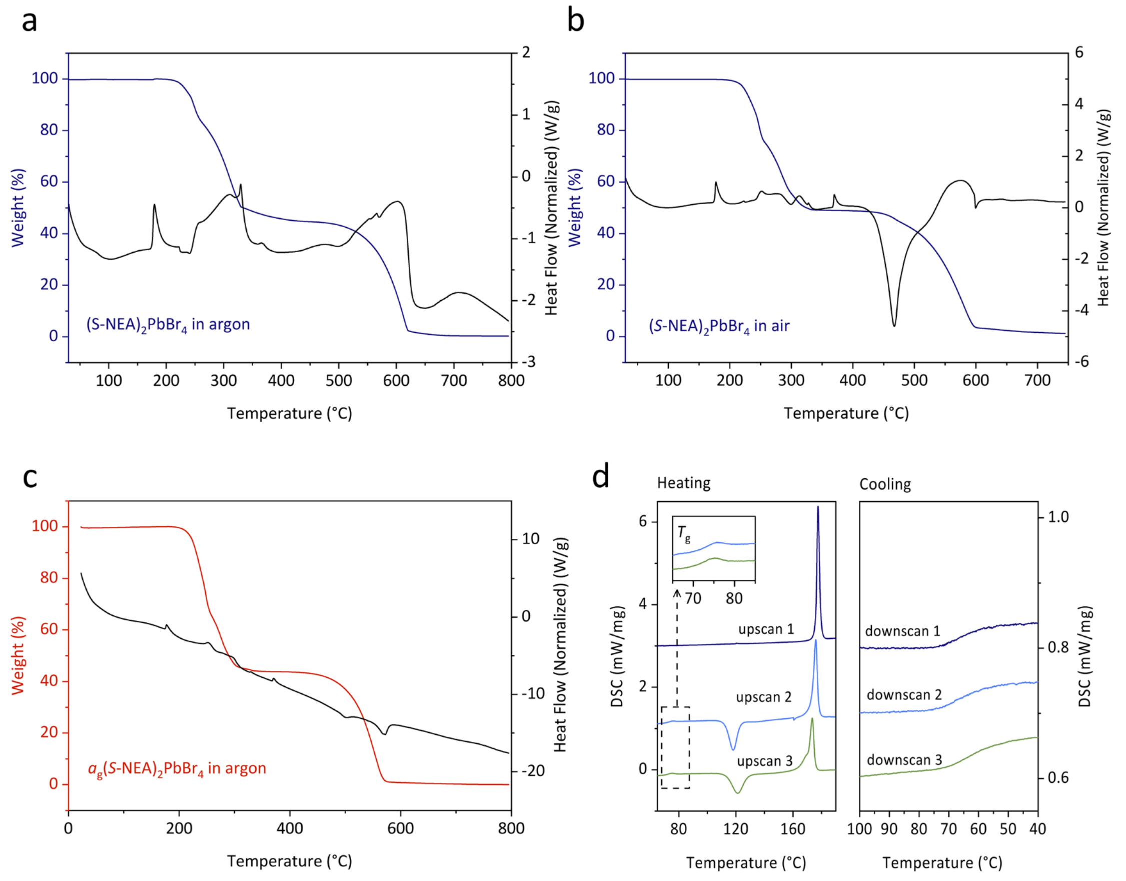

Supplementary Fig. 3. Simultaneous DSC-TGA experiment on (*S*-NEA)$_2$PbBr$_4$. (a) crystalline (*S*-NEA)$_2$PbBr$_4$ in argon, showing a decomposition temperature of *ca*. 220°C and a melting temperature of *ca*. 180°C; (b) crystalline (*S*-NEA)$_2$PbBr$_4$ in air, showing a decomposition temperature of *ca*. 215°C and a melting temperature of *ca*. 178°C; (c) a_g(*S*-NEA)$_2$PbBr$_4$ in argon, showing a decomposition temperature of *ca*. 210°C; (d) Cyclic DSC heating-cooling profiles of crystalline (*S*-NEA)$_2$PbBr$_4$ in argon, demonstrating the reversible crystal-glass transition. The inset shows the glass transition behaviours of the resultant glass after heating-cooling cycles.

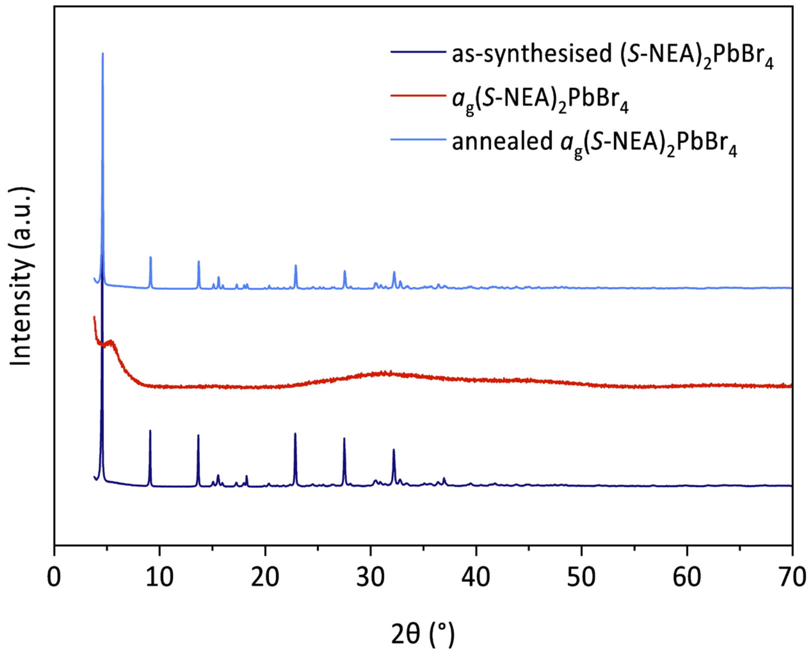

Supplementary Fig. 4. PXRD pattern of the as-synthesised, glassy and recrystallised samples. The intensity of the PXRD peaks for a_g(*S*-NEA)$_2$PbBr$_4$ is amplified by 10 times to highlight the broad features. X-ray wavelength = 1.5418 Å.

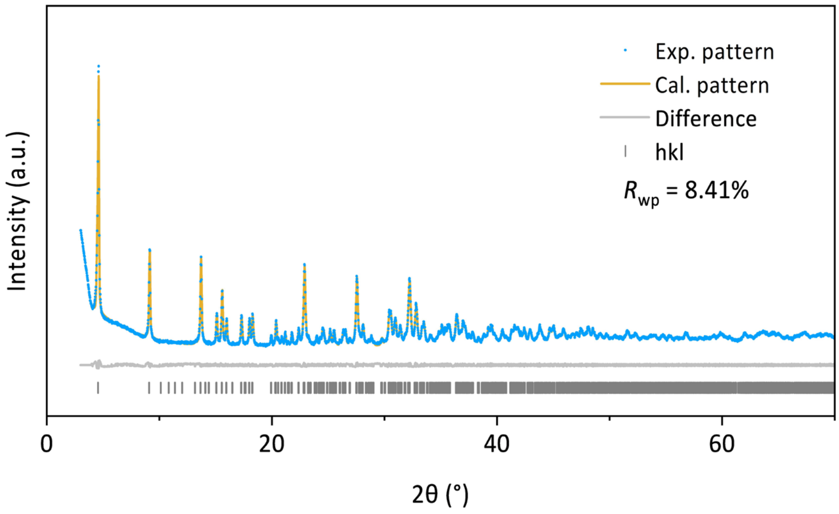

Supplementary Fig. 5. Pawley refinement of the PXRD for recrystallised (S-NEA)$_2$PbBr$_4$, along with patterns simulated from the published Crystallographic Information File (CCDC 2015618)[1]. Experimental data (blue dots), calculated diffraction pattern (yellow line), difference function (grey line) and symmetry-allowed reflections (grey ticks). X-ray wavelength = 1.5418 Å.

Supplementary Table 2.

Crystallographic data from Pawley refinement of the PXRD for recrystallised (S-NEA)$_2$PbBr$_4$.

R_{wp}-value	Space Group	Lattice Parameters	Reported Lattice Parameters[1]
8.41%	$P2_1$	a = 8.7575(8) Å	a = 8.75370 (19) Å
		b = 7.9615(6) Å	b = 7.95502 (16) Å
		c = 19.5020(6) Å	c = 19.5038 (5) Å
		α = 90°	α = 90°
		β = 93.854(7)°	β = 93.806 (2)°
		γ = 90°	γ = 90°

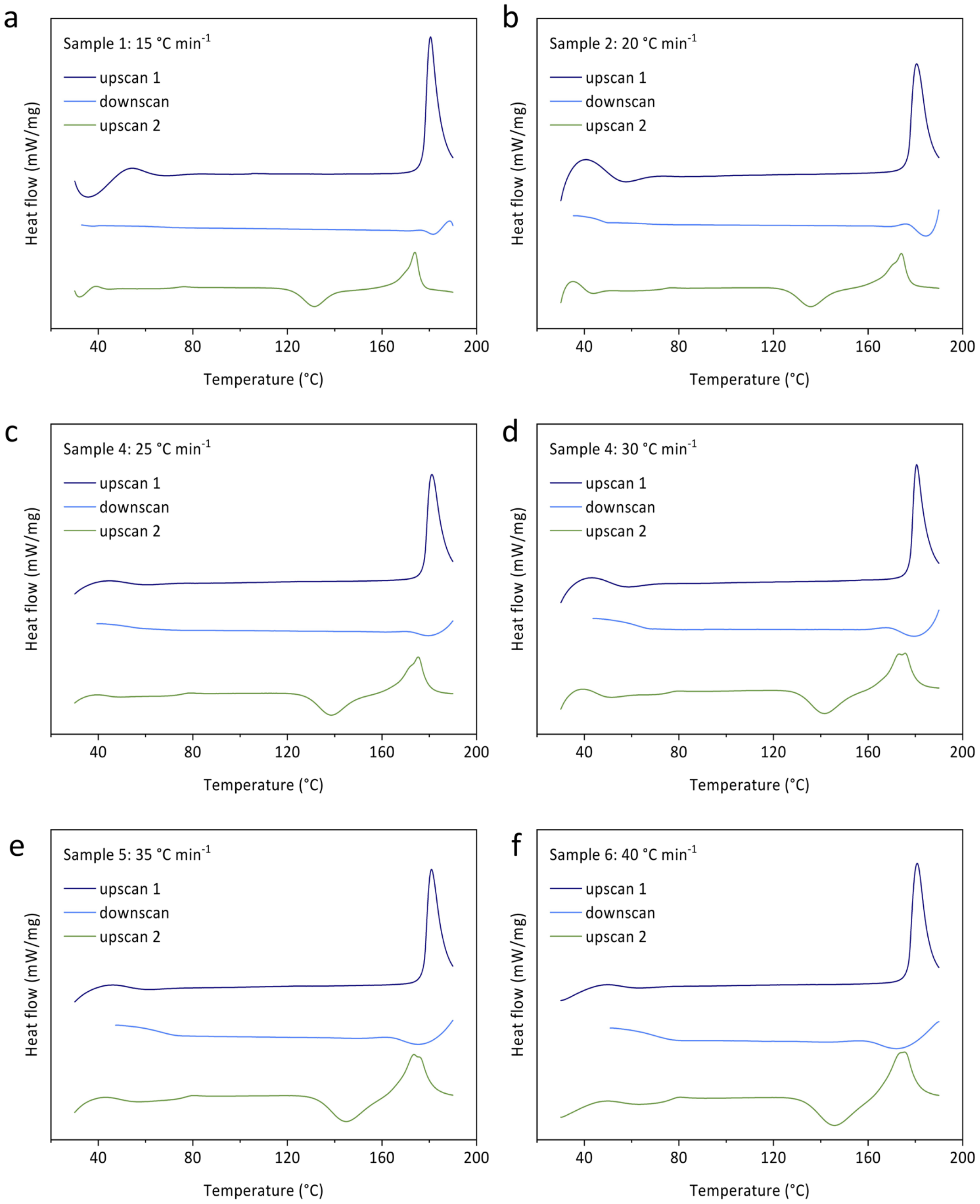

Supplementary Fig. 6. Fragility measurement of the glass-forming liquid (*S*-NEA)$_2$PbBr$_4$. (a-f) Full DSC profiles of the crystalline (*S*-NEA)$_2$PbBr$_4$ samples in argon, showing first-heating, first-cooling, and second-heating scans. The first heating rates for all samples were 40 °C min^{-1}, while different first cooling and second heating rates (q_h) were applied for each sample, including 15 °C min^{-1} for sample 1, 20 °C min^{-1} for sample 2, 25 °C min^{-1} for sample 3, 30 °C min^{-1} for sample 4, 35 °C min^{-1} for sample 5 and 40 °C min^{-1} for sample 6. (continued)

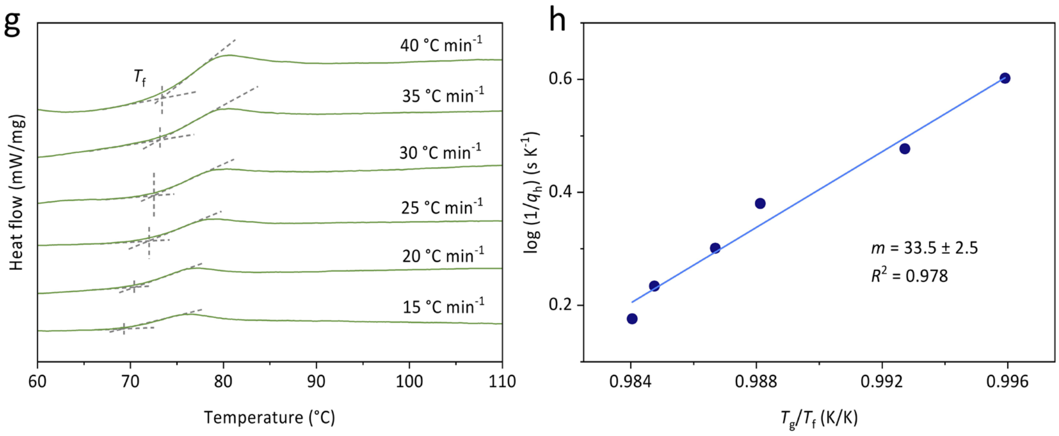

Supplementary Fig. 6. (continued) (g) Comparison of the second heating DSC scans of samples at different q_h ranging from 15 to 40 °C min^{-1}, showing the shift in fictive temperature (T_f) with increasing rate; (h) Extraction of the fragility index (m) from the slope of the linear fit in the plot of log(1/q_h) versus T_g/T_f, where T_g is the standard fictive temperature obtained at a heating rate of 10 °C min^{-1}.

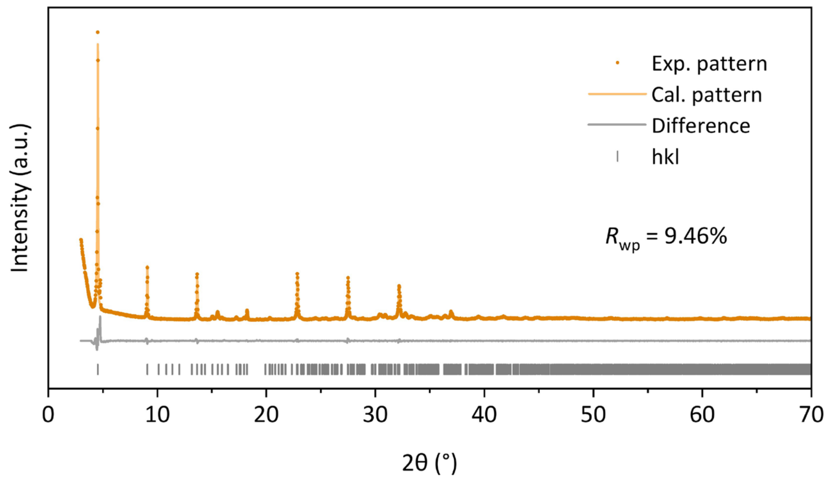

Supplementary Fig. 7. Pawley refinement of the PXRD for crystalline (R-NEA)$_2$PbBr$_4$, along with patterns simulated from the published Crystallographic Information File (CCDC 2015620)[1]. Experimental data (orange dots), calculated diffraction pattern (orange line), difference function (grey line) and symmetry-allowed reflections (grey ticks). X-ray wavelength = 1.5418 Å.

Supplementary Table 3.

Crystallographic data from Pawley refinement of the PXRD for crystalline (R-NEA)$_2$PbBr$_4$.

R_{wp}-value	Space Group	Lattice Parameters	Reported Lattice Parameters[1]
9.46%	$P2_1$	a = 8.7571 (44) Å	a = 8.75686 (18) Å
		b = 7.9646 (38) Å	b = 7.96109 (18) Å
		c = 19.4993 (11) Å	c = 19.5188 (6) Å
		α = 90°	α = 90°
		β = 93.806 (38)°	β = 93.773 (2)°
		γ = 90°	γ = 90°

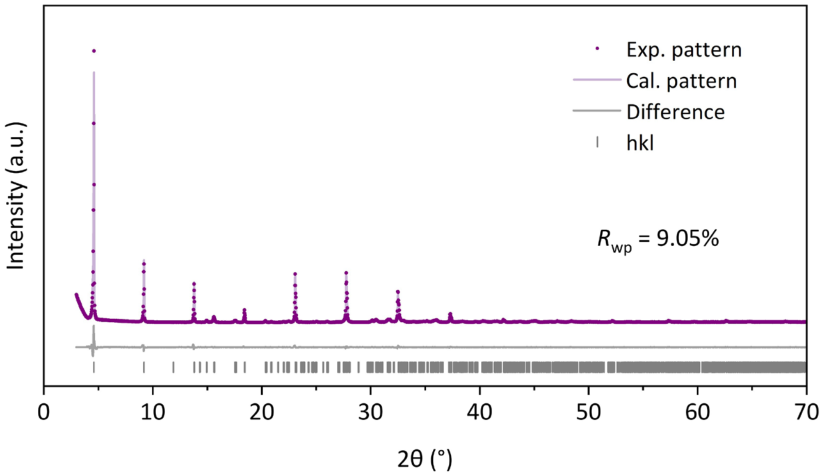

Supplementary Fig. 8. Pawley refinement of the PXRD for crystalline (*rac*-NEA)$_2$PbBr$_4$, along with patterns simulated from the published Crystallographic Information File (CCDC 2015614)[1]. Experimental data (purple dots), calculated diffraction pattern (purple line), difference function (grey line) and symmetry-allowed reflections (grey ticks). X-ray wavelength = 1.5418 Å.

Supplementary Table 4.

Crystallographic data from Pawley refinement of the PXRD for crystalline (*rac*-NEA)$_2$PbBr$_4$.

R_{wp}-value	Space Group	Lattice Parameters	Reported Lattice Parameters[1]
		a = 19.2789(3) Å	*a* = 19.2528(9) Å
		b = 8.0848(2) Å	*b* = 19.2528(9) Å
		c = 8.7393(2) Å	*c* = 8.7280(5) Å
9.05%	$P2_1/c$	α = 90°	α = 90°
		β = 90.583(35)°	β = 90.281(3)°
		γ = 90°	γ = 90°

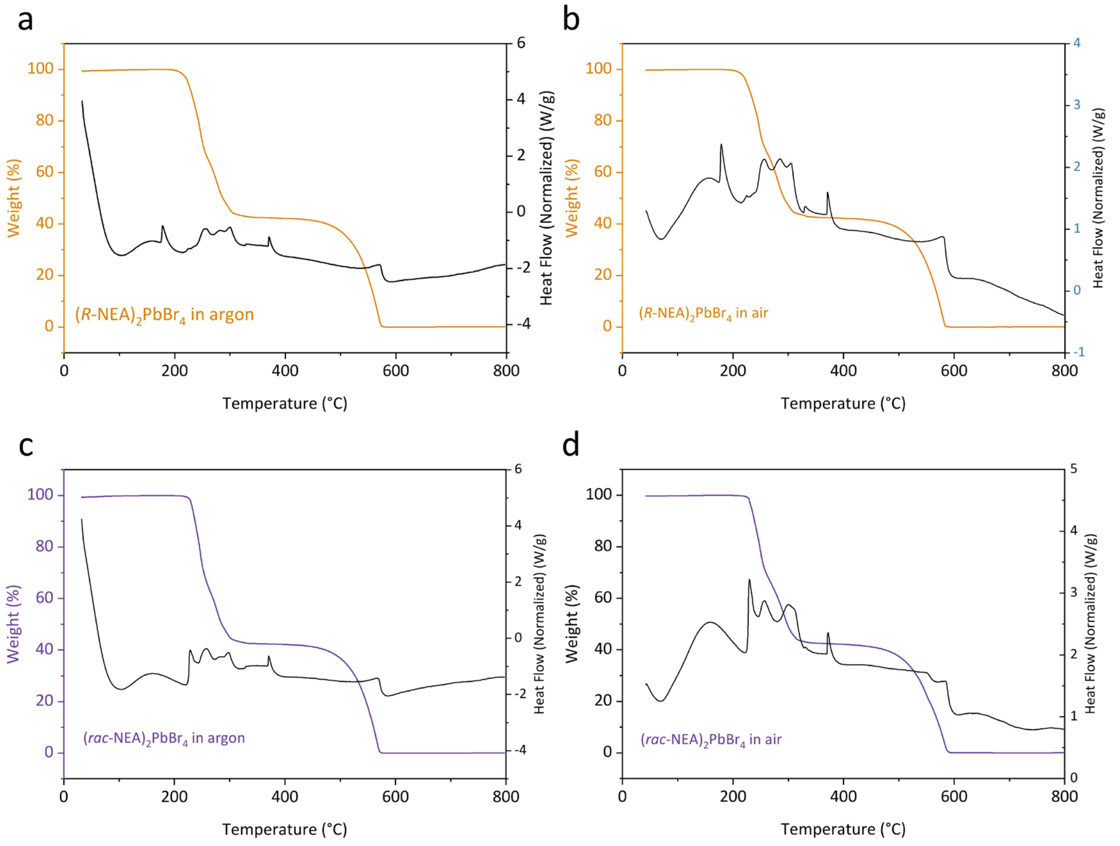

Supplementary Fig. 9. Simultaneous DSC-TGA experiment on (*R*-NEA)$_2$PbBr$_4$ and (*rac*-NEA)$_2$PbBr$_4$. (a) crystalline (*R*-NEA)$_2$PbBr$_4$ in argon, showing a decomposition temperature of *ca*. 216°C and a melting temperature of *ca*. 178°C; (b) crystalline (*R*-NEA)$_2$PbBr$_4$ in air, showing a decomposition temperature of ca. 215°C and a melting temperature of ca. 179°C; (c) crystalline (*rac*-NEA)$_2$PbBr$_4$ in argon, showing a decomposition temperature of *ca*. 225°C and a melting temperature of *ca*. 230°C; (d) crystalline (*rac*-NEA)$_2$PbBr$_4$ in air, showing a decomposition temperature of *ca*. 227°C and a melting temperature of *ca*. 230°C.

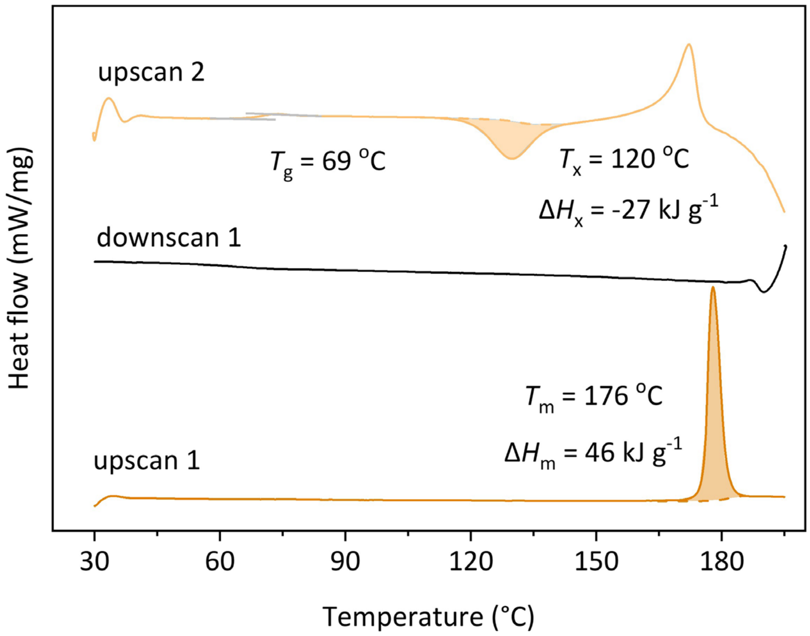

Supplementary Fig. 10. DSC experiment of (R-NEA)$_2$PbBr$_4$ in argon. The heating rate and cooling rate were both 10 °C min^{-1}. Melting of crystalline (R-NEA)$_2$PbBr$_4$ happened at T_m = *ca*. 176°C, with a difference in enthalpy of melting (ΔH_m) of *ca*. 46 kJ g^{-1}. The glass transition temperature of a_g(R-NEA)$_2$PbBr$_4$ was observed to be *ca*. 69°C, and the crystallisation happened at T_x = *ca*. 120°C, with a difference in enthalpy of crystallisation (ΔH_x) of *ca*. -27 kJ g^{-1}.

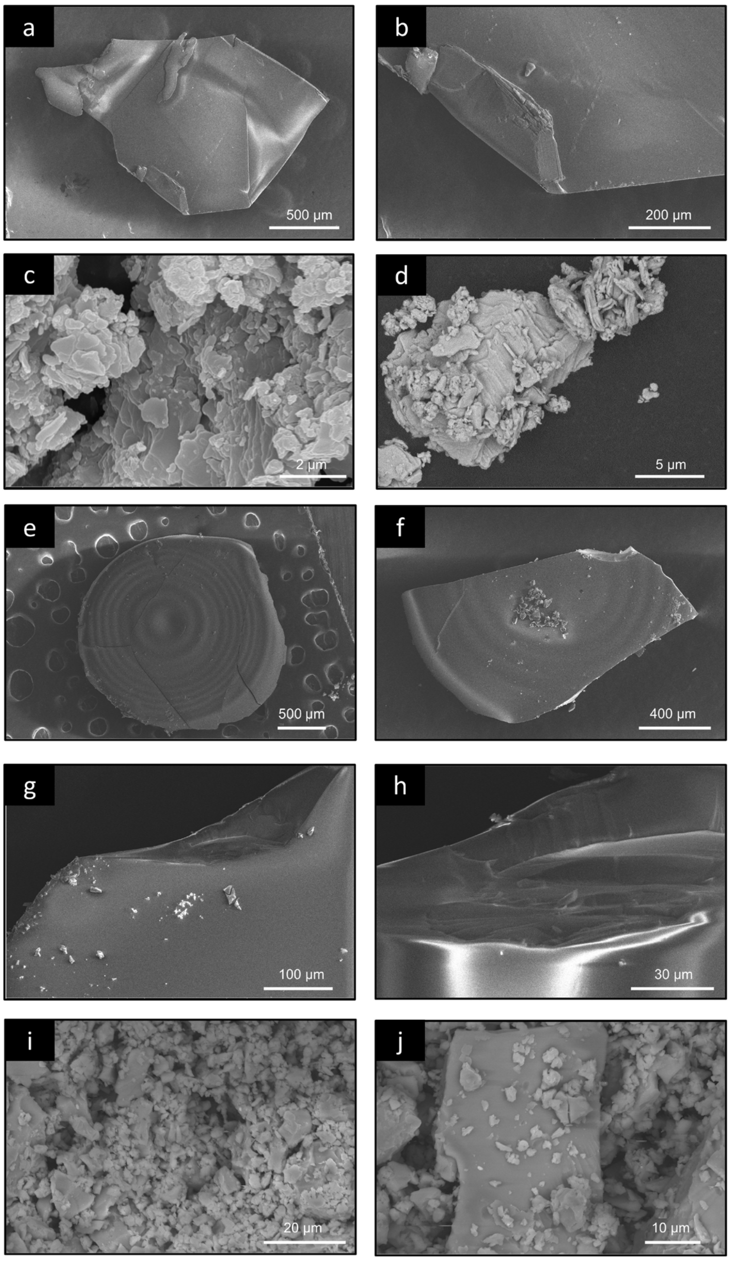

Supplementary Fig. 11. Scanning electron microscopy images of (*S*-NEA)$_2$PbBr$_4$ crystal and glass. (a-b) As-synthesised bulk crystals; (c-d) ground crystalline powders; (e-f) melt-quenched bulk glass; (g-h) smooth edge of glass flakes; (i-j) ground glassy powders.

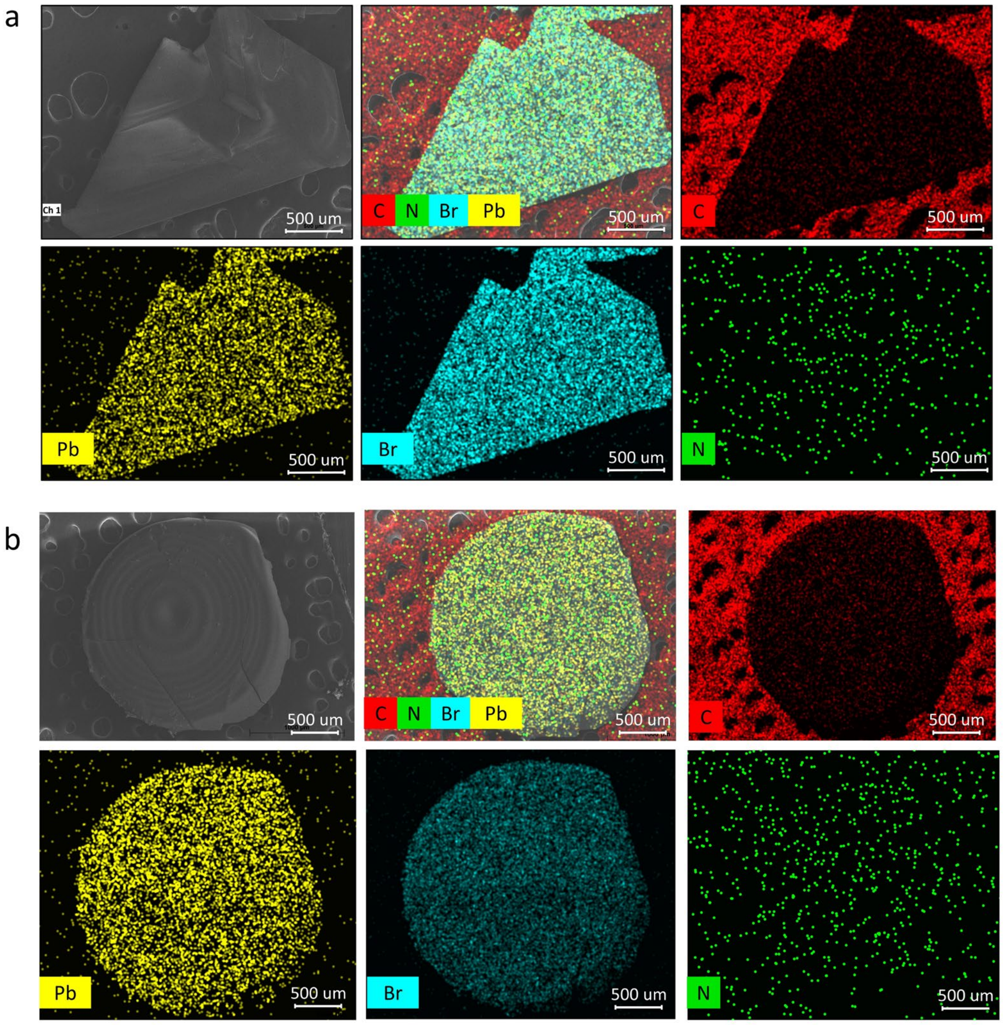

Supplementary Fig. 12. SEM-EDS elemental mapping of (a) (*S*-NEA)$_2$PbBr$_4$ and (b) a_g(*S*-NEA)$_2$PbBr$_4$. The element mapping show the homogeneous distribution of Pb (yellow), Br (blue), C (red) and N (green) atoms throughout the entire crystal and the glass at the millimeter scale.

Supplementary Table 5. Elemental analysis of (*S*-NEA)$_2$PbBr$_4$ and a_g(*S*-NEA)$_2$PbBr$_4$ calculated from SEM-EDS data.

Substance	C* (wt %)	N* (wt %)	Br (wt %)	Pb (wt %)	Br : Pb (molar ratio)
Theoretical	33.05	3.21	36.68	23.78	4.0
(*S*-NEA)$_2$PbBr$_4$	78.22	2.19	11.66	7.93	3.81
a_g(*S*-NEA)$_2$PbBr$_4$	80.98	2.48	9.71	6.83	3.69

* The concentrations of C and N are underestimated due to the lower sensitivity of EDS for lighter elements.

Supplementary Table 6. CHN microanalysis of (S-NEA)$_2$PbBr$_4$ and a_g(S-NEA)$_2$PbBr$_4$.

Substance		Weight taken (mg)	C (%)	H (%)	N (%)
Theoretical		-	33.08	3.24	3.22
(S-NEA)$_2$PbBr$_4$	Test 1	2.1766	33.80	3.27	3.29
	Test 2	1.7610	33.79	3.25	3.26
	Mean (ESD)	-	33.80 (0.01)	3.26 (0.01)	3.28 (0.02)
a_g (S-NEA)$_2$PbBr$_4$	Test 1	1.3751	32.28	3.06	3.10
	Test 2	1.4905	32.25	3.06	3.13
	Mean (ESD)	-	32.27 (0.02)	3.26 (0.00)	3.12 (0.02)

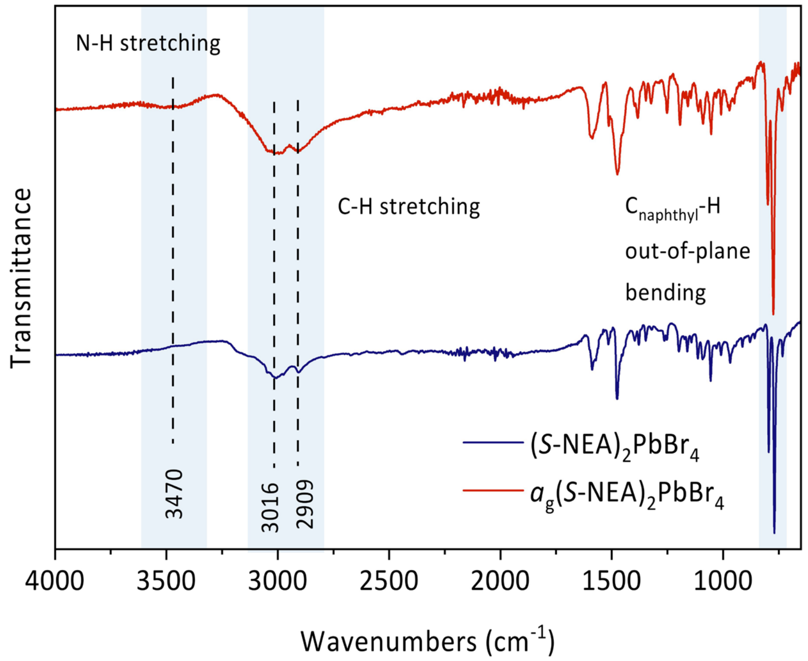

Supplementary Fig. 13. Fourier-transform infrared (FTIR) spectra of (S-NEA)$_2$PbBr$_4$ and a_g(S-NEA)$_2$PbBr$_4$. Key vibrational features are assigned as follows: N-H stretching near 3470 cm^{-1}, C-H stretching (both aromatic and aliphatic) around 3016 and 2909 cm^{-1}, and out-of-plane bending of aromatic C-H from the naphthyl moiety near 750 cm^{-1}. The increased intensity of the 3470 cm^{-1} band in the glass is attributed to a more weakly hydrogen-bonded N-H environment, likely arising from increased structural disorder upon vitrification.

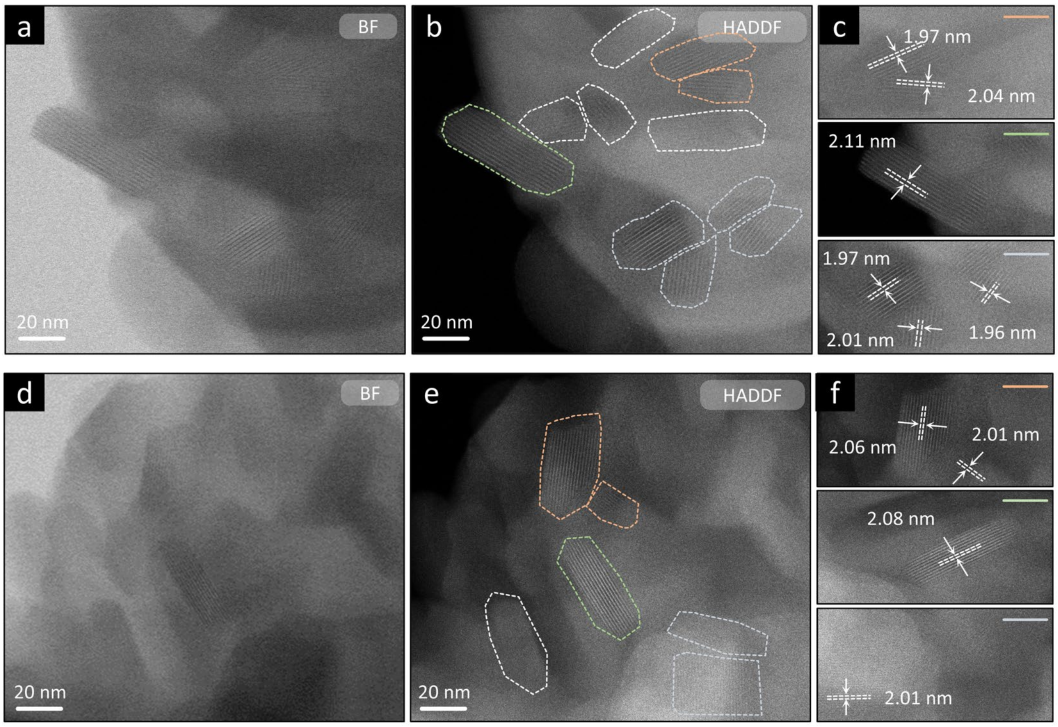

Supplementary Fig. 14. STEM images of crystalline (*S*-NEA)$_2$PbBr$_4$. (a, d) Bright-field (BF) STEM images showing lattice-fringe-resolved regions in the crystalline domains. (b, e) High-angle annular dark-field (HAADF) STEM images of the same areas, with selected crystalline domains outlined in coloured dashed lines. (c, f) Corresponding *d*-spacing measurements from the highlighted regions in (b) and (e), confirming periodic lattice fringes with interplanar spacings of approximately 2 nm. Scale bar, 20 nm.

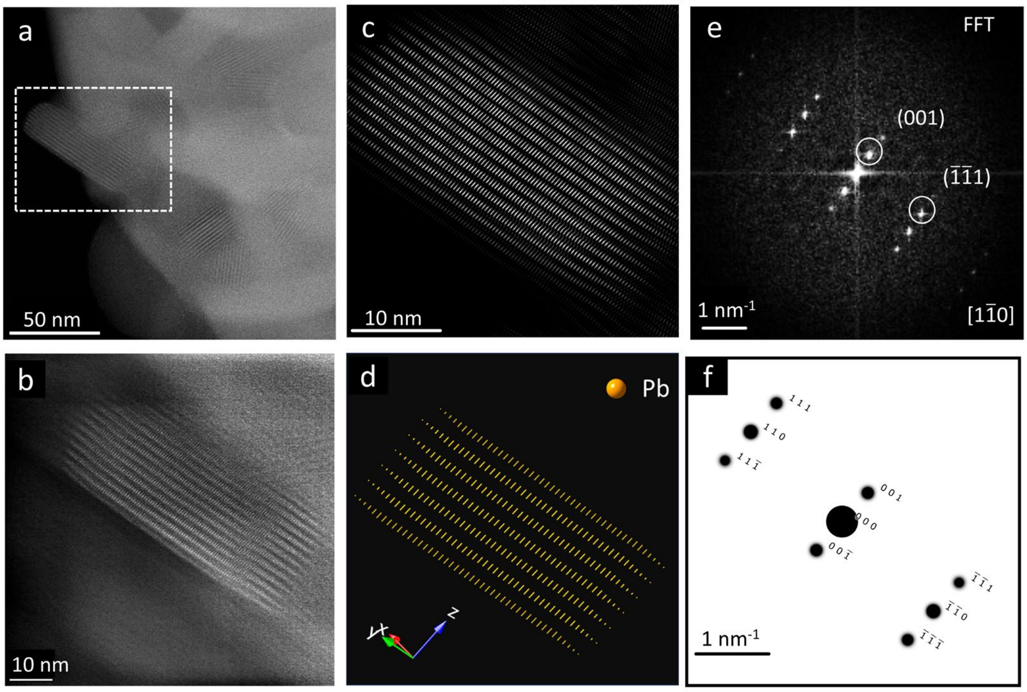

Supplementary Fig. 15. HAADF-STEM analysis of crystalline (*S*-NEA)$_2$PbBr$_4$. (a) HAADF image showing multiple crystalline domains; the white box marks the region further analysed in (b-e). (b) Filtered image of a selected crystalline domain from (a), processed using radial Wiener filtering. (c) Atomic-resolution image obtained from further processing of the region shown in (b), revealing atomic-scale lattice fringes. (d) Simulated atomic model of Pb atoms, corresponding to the observed lattice orientation in (c). (e) FFT of the lattice-resolved image in (c), indexed to the $[1\bar{1}0]$ zone axis, with (001) and $(\bar{1}\bar{1}1)$ reflections circled. (f) Simulated electron diffraction pattern for the $[1\bar{1}0]$ zone axis, showing good agreement with the experimental FFT in (e).

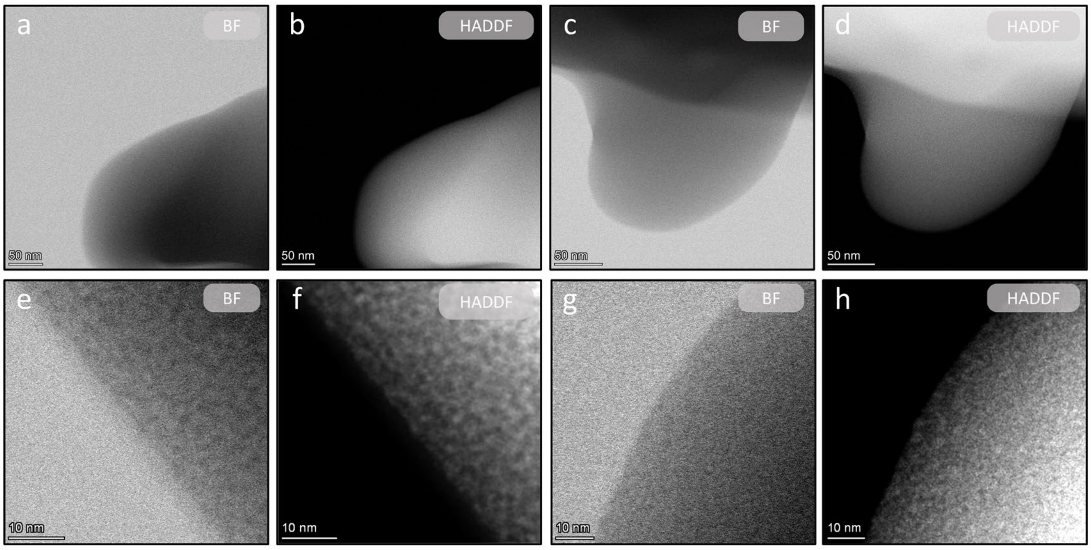

Supplementary Fig. 16. STEM images of $\boldsymbol{a_g}$(*S*-NEA)$_2$PbBr$_4$. (a, c, e, g) Bright-field (BF) STEM images acquired at different magnifications. (b, d, f, h) Corresponding high-angle annular dark-field (HAADF) STEM images of the same regions. At both low (a-d) and high (e-h) magnifications, the glass sample exhibits diffuse, non-periodic contrast with no discernible lattice fringes, indicating a lack of long-range structural order. The uniformity in contrast across the sample suggests a homogeneously amorphous phase at the nanoscale and may indicate the presence of short-range ordering around Pb atoms.

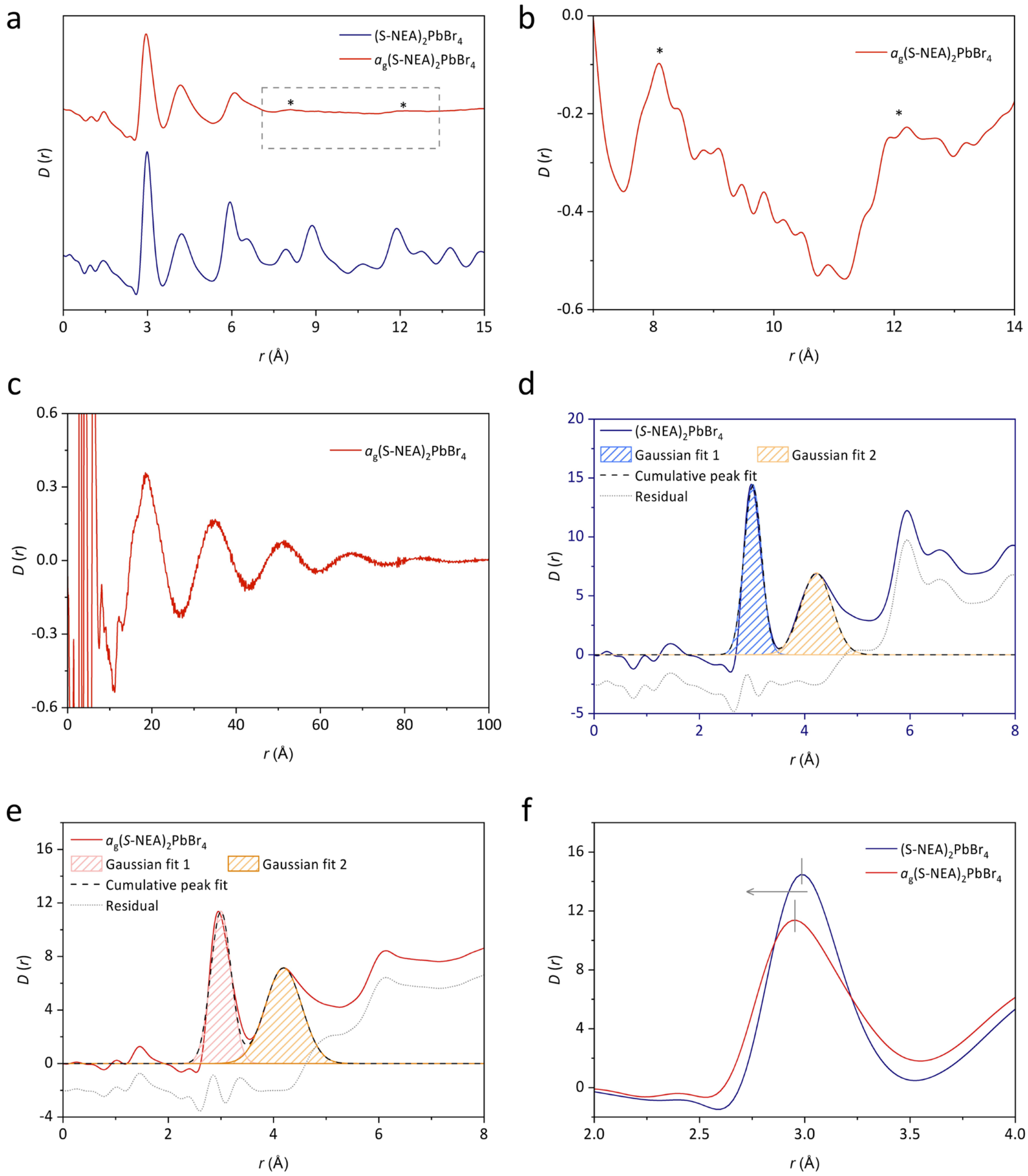

Supplementary Fig. 17. Ambient-temperature X-ray pair distribution functions. (a) Comparison between crystalline and glassy (S-NEA)$_2$PbBr$_4$ in a r-range of 0-15 Å, where asterisks (*) denote the weak intermediate-r peaks in glassy-phase PDF. Expanded view of the PDF of the glassy phase (b) in a r-range of 7-14 Å, highlighting the intermediate-r peaks at approximately 8 Å and 12 Å; (c) in a r-range of 0-100 Å, highlighting the quasiperiodic oscillations in the extended-r range. Peak fitting of the first and second peak in the (d) crystalline- and (e) glassy-phase PDF (baseline extracted) using Gaussian functions. For clarity, the residual lines in panels (d) and (e) were offset by -2.5 and -2, respectively. For detailed fitting parameters, see Supplementary Table 7. (f) Pb-Br peak shift from crystalline- to glassy-phase PDF. Data were collected using the I15-1 beamline at the Diamond Light Source (Oxfordshire, UK) with a wavelength of λ = 0.161669 Å.

Supplementary Table 7. Fitting parameters of the Gaussian fits for RT-PDF data.

Fitting parameters		$(S\text{-NEA})_2PbBr_4$ phase	
		crystalline	glassy
Slope of Baseline D(r) = -4πρ*r		-1.0942	
Peak value		2.99	2.95
Gaussian fit 1	Area	6.01	5.51
	FWHM	0.39	0.45
	Center	3.01	2.99
	Amplitude	14.83	11.38
	$CN_{\text{Pb-Br}}$	6.00	5.50
Gaussian fit 2	Area	5.10	6.19
	FWHM	0.69	0.81
	Center	4.22	4.19
	Amplitude	6.94	7.15

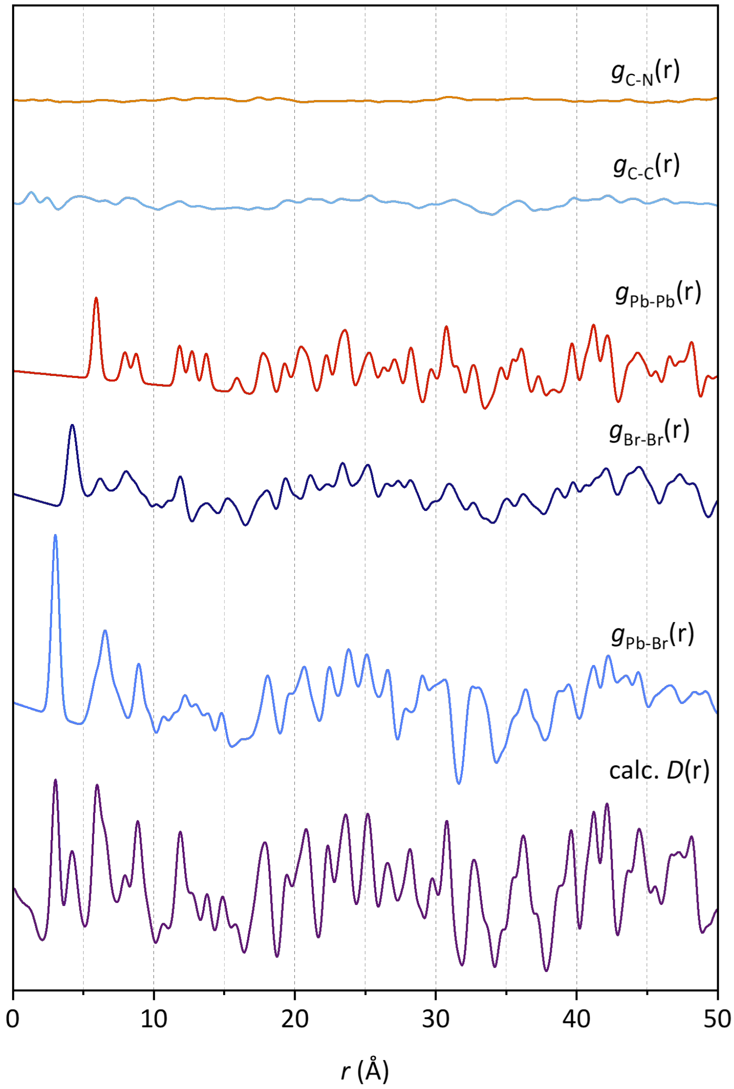

Supplementary Fig. 18. X-ray calculated total and partial pair distribution functions for (*S*-NEA)$_2$PbBr$_4$, which were generated from the crystal structure using PDFgui (CCDC 2015618)[1]. The X-ray form factors of the constituent C, N and H atoms are so small compared to those of Pb and Br atoms that the contribution of the organic cation (S-NEA)$^+$ to the PDF pattern can almost be omitted.

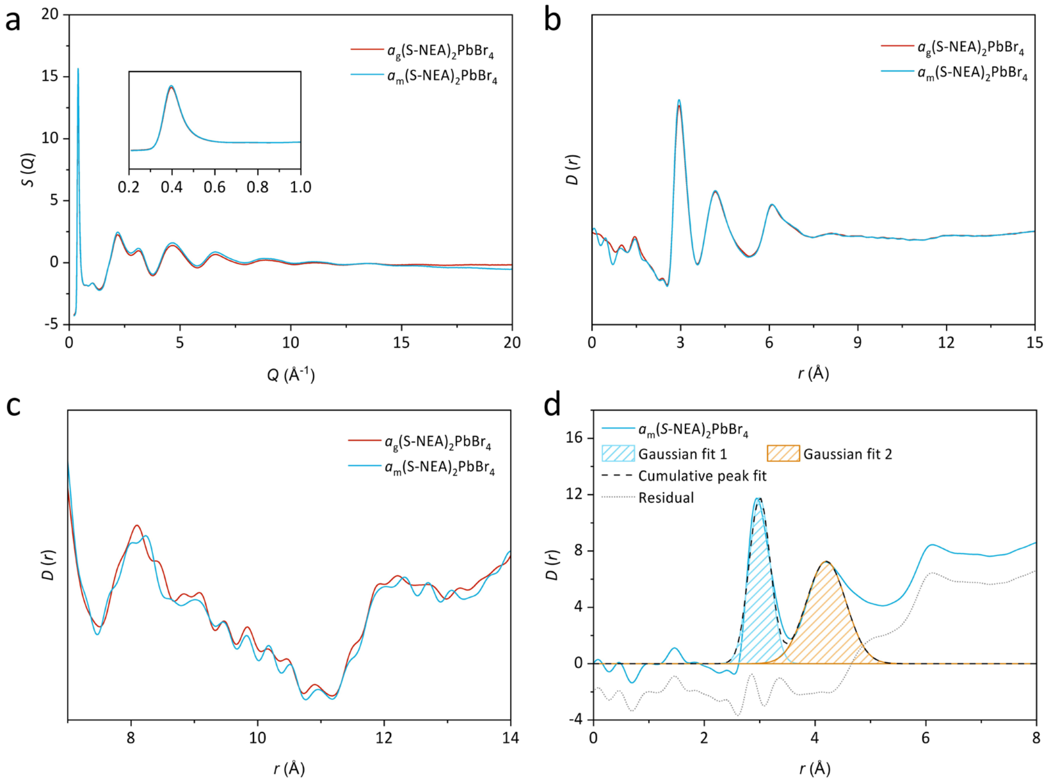

Supplementary Fig. 19. Total scattering data for ball-milled glass a_m(*S*-NEA)$_2$PbBr$_4$, obtained from our previous study[2] and processed using the same condition as those for the melt-quenched glass a_g(*S*-NEA)$_2$PbBr$_4$. (a) Comparison of the structure factor *S*(Q) between a_g(*S*-NEA)$_2$PbBr$_4$ and a_m(*S*-NEA)$_2$PbBr$_4$, with the inset showing the corresponding low-*Q* feature. Comparison of the pair distribution functions *D*(*r*) between a_g(*S*-NEA)$_2$PbBr$_4$ and a_m(*S*-NEA)$_2$PbBr$_4$, in the range of (b) 0-15 Å and (c) 7-14 Å, highlighting their similarity in the short-range and intermediate-range ordering. (d) Gaussian peak fitting of the pair distribution function for a_m(*S*-NEA)$_2$PbBr$_4$. The residual line was offset by -2 for clarity. The integral of the Pb-Br peak were calculated to be 5.62, corresponding to an average Pb coordination number of *ca*. 5.6 for the ball-milled glass. This is comparable to that of the melt-quenched glass, which has a value of *ca*. 5.5.

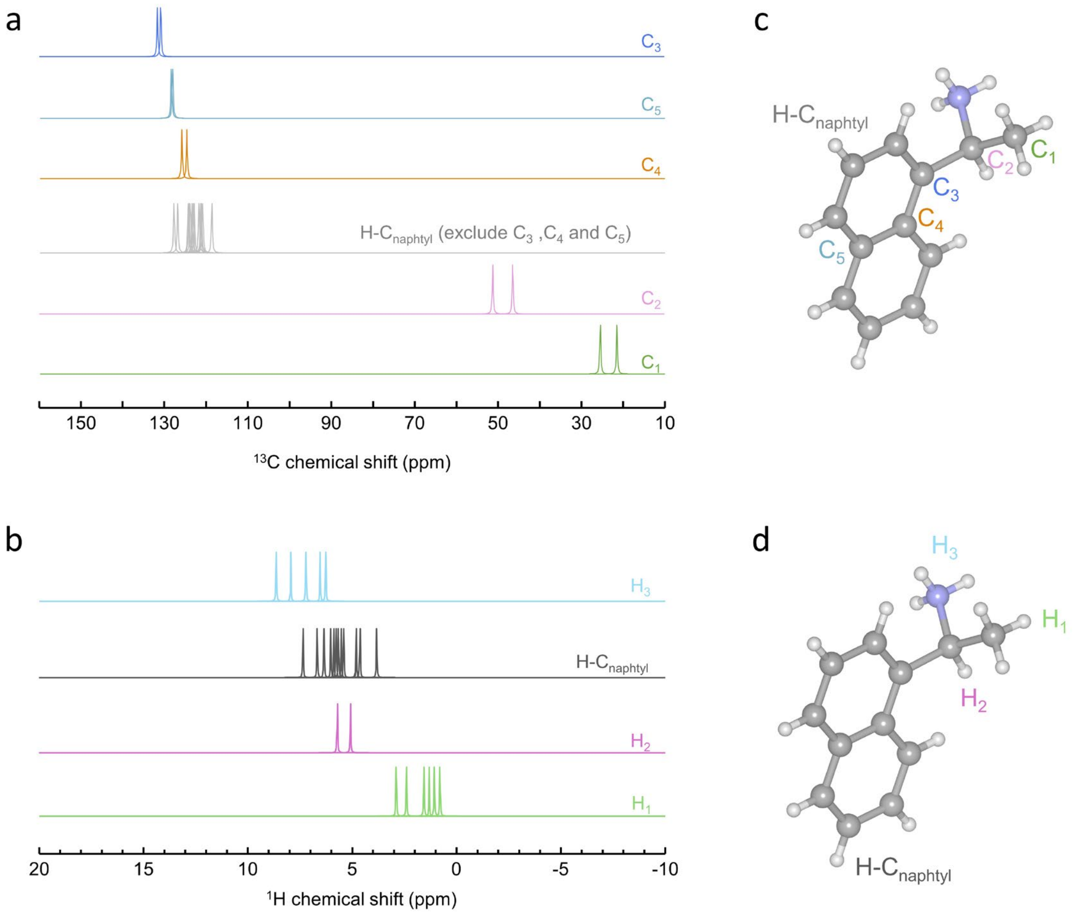

Supplementary Fig. 20. DFT-calculated NMR spectra and atom assignments for (*S*-NEA)$_2$PbBr$_4$. (a) Simulated ^{13}C and (b)^{1}H chemical shifts with line assignments to specific carbon and hydrogen atoms in the organic cation. (c, d) Molecular structure of (*S*)-(–)-1-(1-naphthyl)ethylammonium (*S*-NEA)$^+$, with corresponding carbon (c) and proton (d) labels used in the spectral assignments.

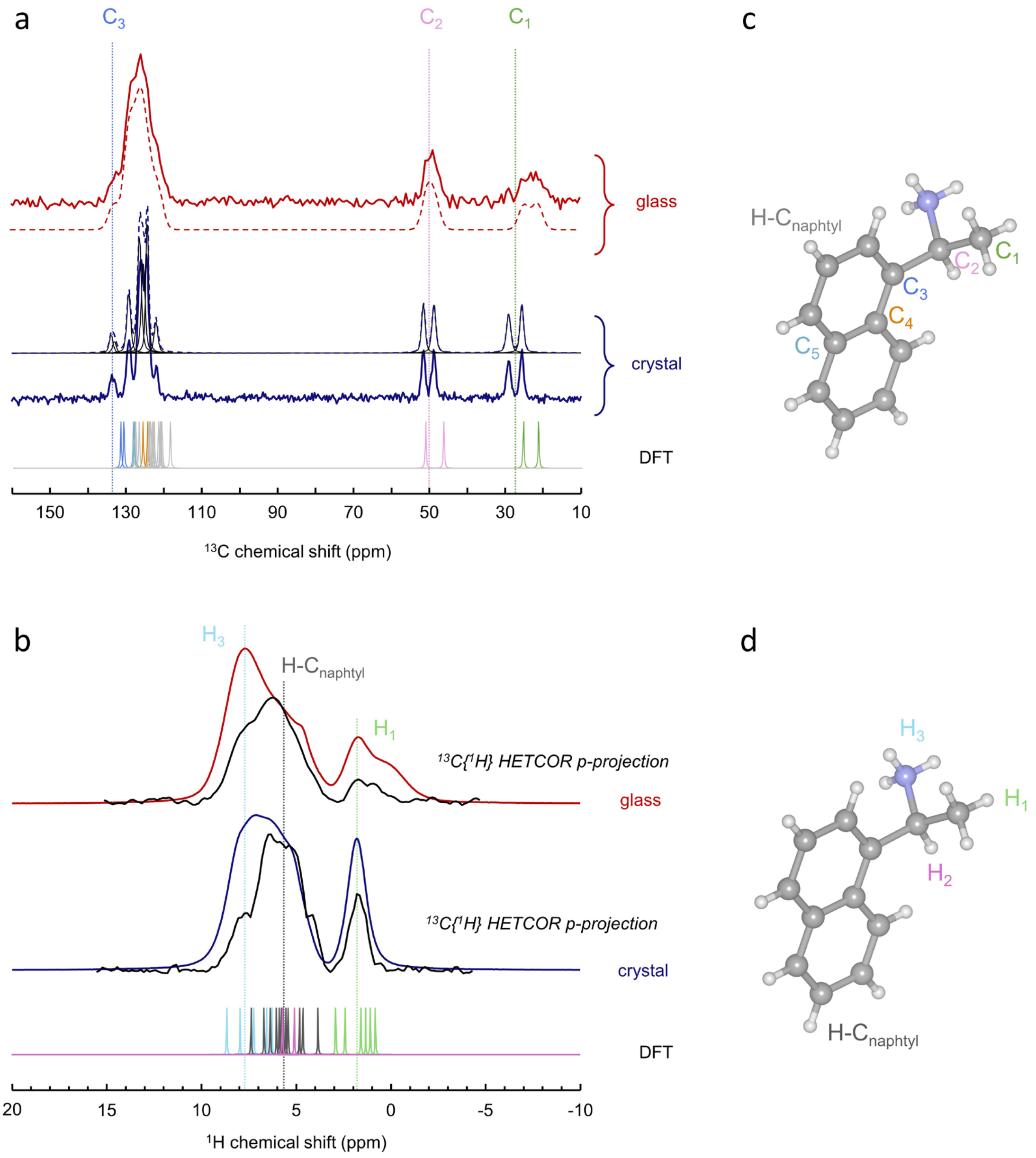

Supplementary Fig. 21. High-speed high-field MAS NMR spectra. (a) Experimental ^{13}C and (b) ^{1}H spectra of crystalline (*S*-NEA)$_2$PbBr$_4$ (blue solid line, middle) and glassy a_g(*S*-NEA)$_2$PbBr$_4$ (red solid line, top), along with the corresponding DFT calculations (bottom). In (a), simulated ^{13}C spectra for crystal (blue dashed line) and glass (red dashed line), as well as individual simulation components (black solid line), are vertically offset for clarity. Vertical dotted lines indicate the theoretical chemical shifts for different carbon atoms and protons in the organic cation. Structure of the organic cation, (*S*)-(−)-1-(1-naphthyl)ethylammonium (*S*-NEA)$^+$, along with (c) carbon and (d) proton labels. In (b), black solid lines denote the ^{1}H projections from the two-dimensional $^{13}C\{^{1}H\}$ HETCOR spectra.

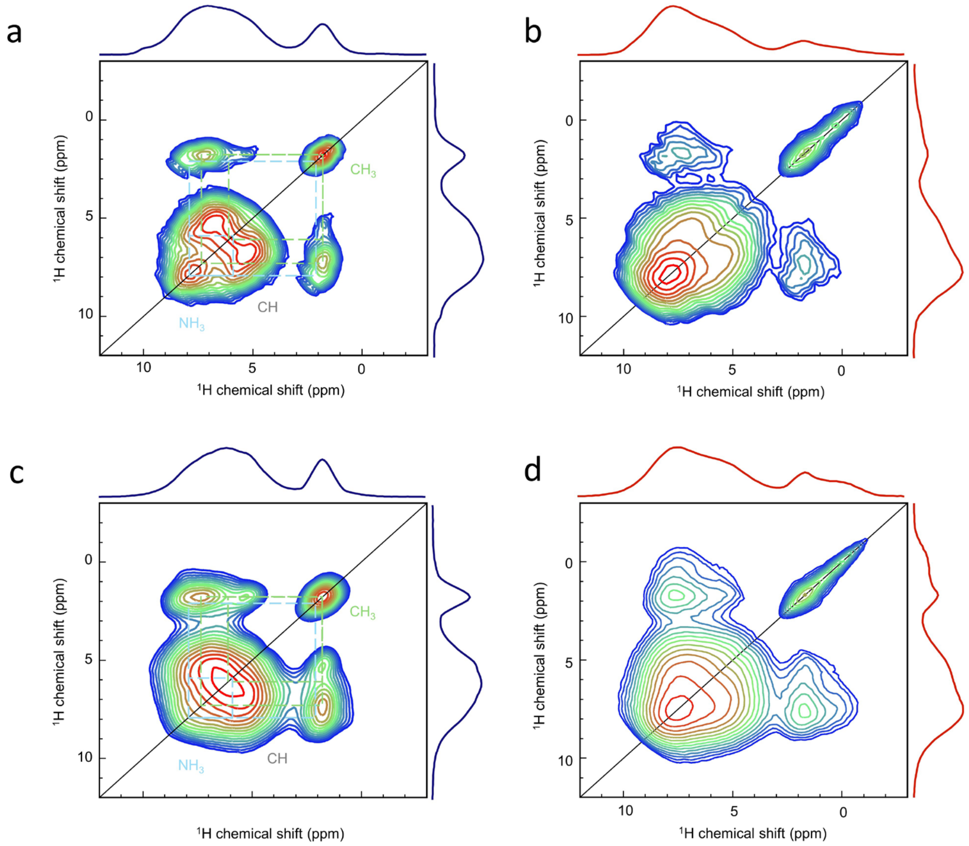

Supplementary Fig. 22. High-speed high-field MAS NMR $^1H\{^1H\}$ double-quantum / single-quantum (DQ/SQ) correlation spectra for (a, c) crystalline (*S*-NEA)$_2$PbBr$_4$ and (b, d) a_g(*S*-NEA)$_2$PbBr$_4$. In (a) and (b), a D-H^3 sequence is used, and the spectra are sheared and symmetrized. In (c) and (d), a SR4$^2_1$ sequence is used, and the spectra are sheared and symmetrized.

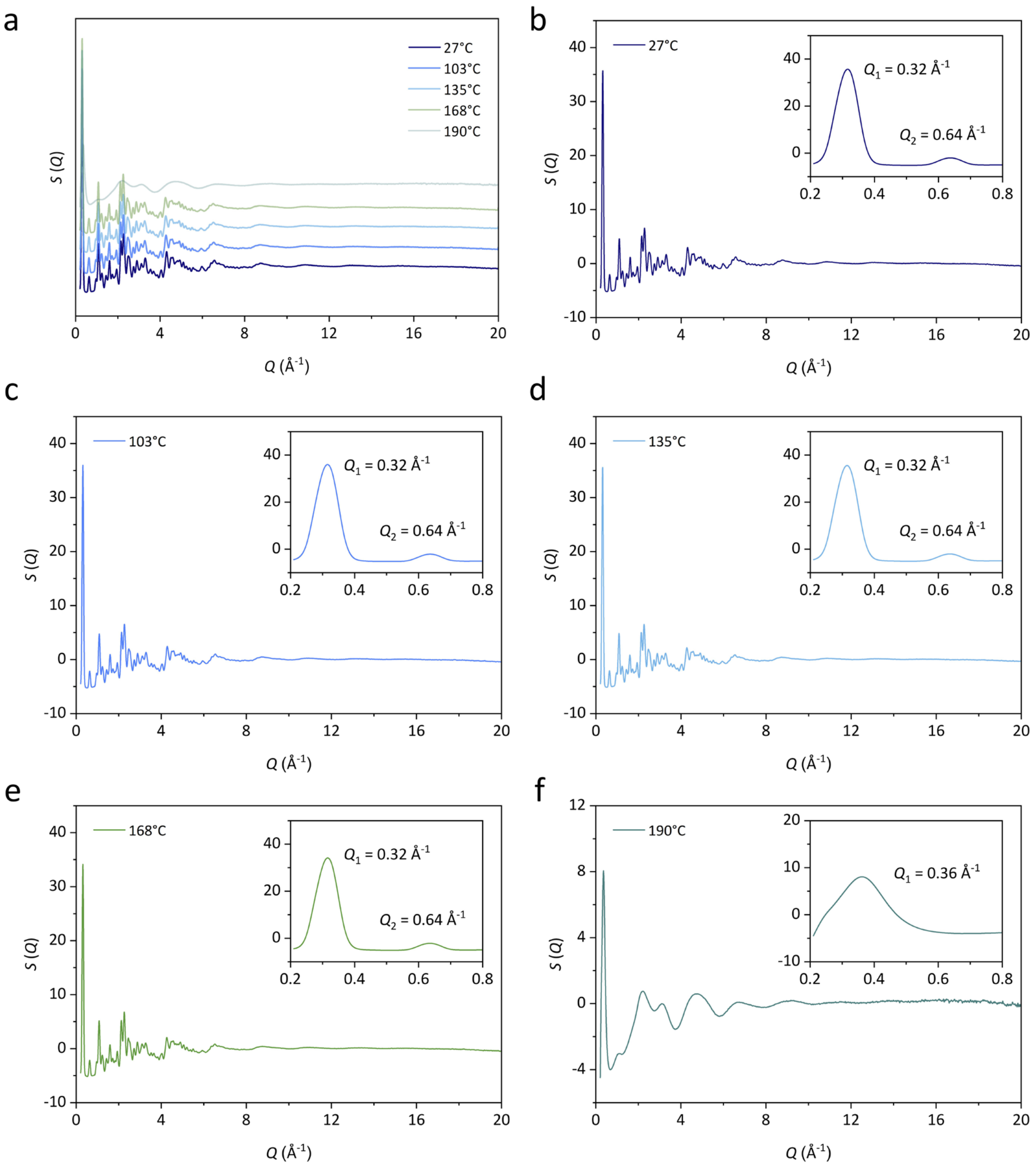

Supplementary Fig. 23. Variable-temperature X-ray total scattering. (a) Structure factor *S*(Q) of crystalline (*S*-NEA)$_2$PbBr$_4$ upon heating from 27°C to 190°C. Individual structure factor *S*(Q) obtained at different temperature point: (b) 27°C, (c) 103°C, (d) 135°C, (e) 168°C and (f) 190°C. Insets show the corresponding low-*Q* features. Data were collected using the PETRA III P02.1 beamline at DESY (Hamburg, Germany) with a wavelength of λ = 0.20734 Å.

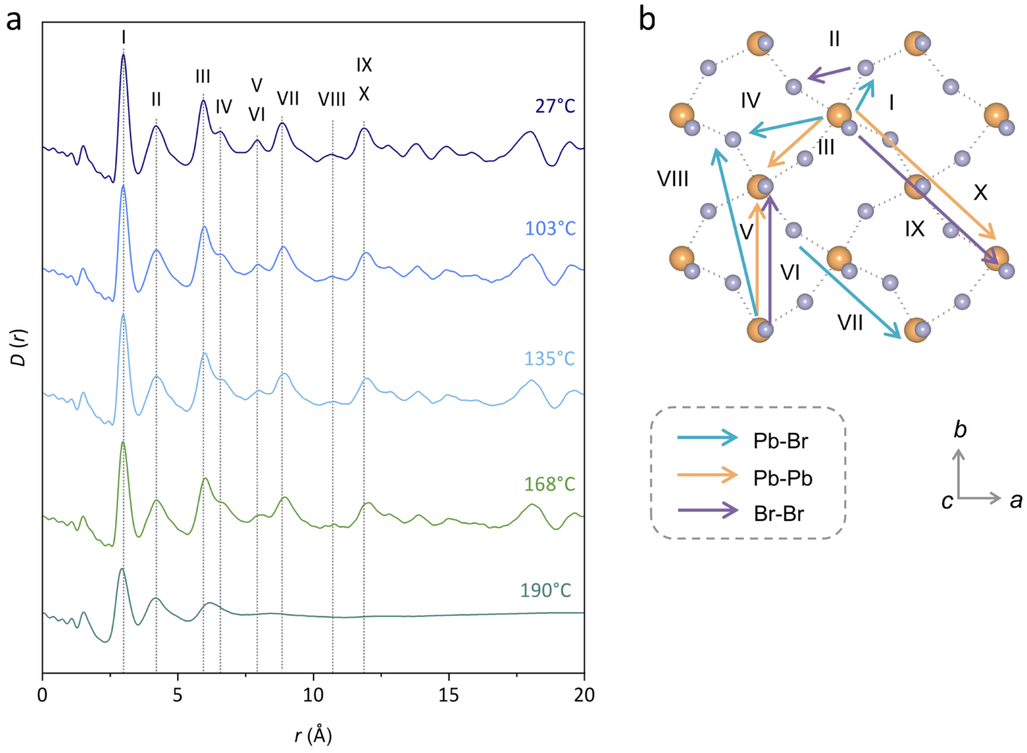

Supplementary Fig. 24. Variable-temperature X-ray pair distribution functions. (a) $D(r)$ of crystalline (S-NEA)$_2$PbBr$_4$ upon heating from 27°C to 190°C, with labelled peaks. (b) The scheme for peak assignment, including the atom pairs that predominately contribute to the PDF intensity. The contribution from atoms pairs containing C, N, H has been omitted as the X-ray form factors of those constituent are so small compared to those of Pb (orange) and Br (purple) atoms.

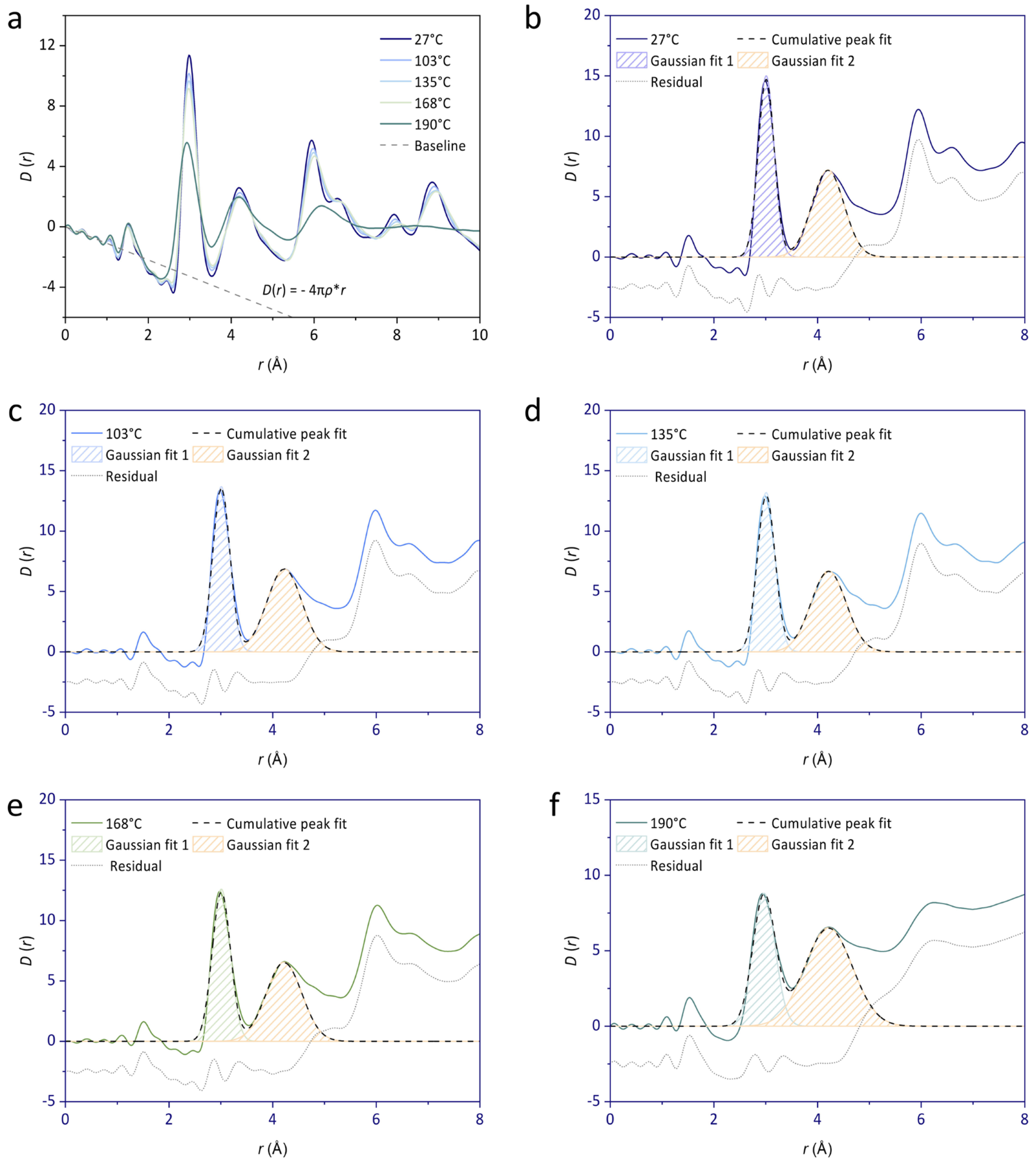

Supplementary Fig. 25. Gaussian peak fitting of variable-temperature X-ray pair distribution function data. (a) $D(r)$ of crystalline (S-NEA)$_2$PbBr$_4$ during heating from 27°C to 190°C. Individual $D(r)$ curve of crystalline (S-NEA)$_2$PbBr$_4$ upon heating at (b) 27°C, (c) 103°C, (d) 135°C, (e) 168°C and (f) 190°C, with Gaussian peak fitting. The residual lines in panels (b-f) were offset by -2.5 for clarity. For detailed fitting parameters, see Supplementary Table 8.

Supplementary Table 8. Fitting parameters of the Gaussian fits for VT-PDF data.

Fitting parameters		Temperature (°C)				
		27	103	135	168	190
Slope of Baseline $D(r) = -4\pi\rho^{*}r$		-1.0942				
Peak value		2.99	2.98	2.98	2.97	2.93
Gaussian Fit 1	Area	5.73	5.61	5.55	5.48	4.73
	FWHM	0.36	0.39	0.40	0.41	0.50
	Center	3.01	3.01	3.01	3.01	2.97
	Amplitude	15.00	13.68	13.19	12.60	8.81
	$CN_{Pb\text{-}Br}$	6.00	5.87	5.81	5.73	4.95
Gaussian Fit 2	Area	5.60	5.80	5.70	5.65	7.40
	FWHM	0.73	0.79	0.80	0.81	1.06
	Center	4.20	4.22	4.21	4.22	4.21
	Amplitude	7.21	6.87	6.69	6.55	6.56

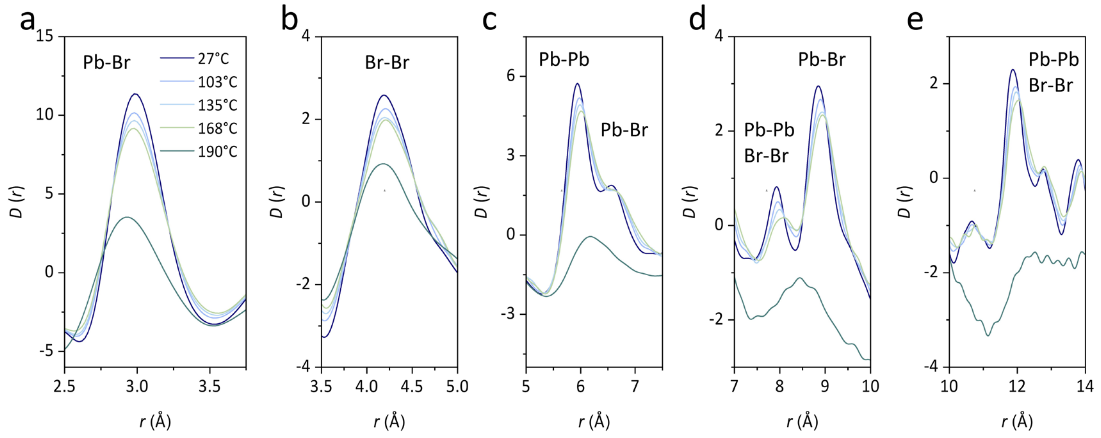

Supplementary Fig. 26. Individual peak analysis of variable-temperature X-ray pair distribution function data. Pair distribution functions *D*(r) of (*S*-NEA)$_2$PbBr$_4$ upon heating from 27°C to 190°C in a r-range of (a) 2.50-3.75 Å, (b) 3.5-5 Å, (c) 5-7.5 Å, (d) 7-10 Å and (e) 10-14 Å, highlighting the different correlations as labelled. Liquid-phase PDFs at 190°C offset for clarity. In (d) and (e), liquid-phase *D*(*r*) at 190°C was amplified by a factor of 5 and 10, to highlight the weak peaks, respectively.

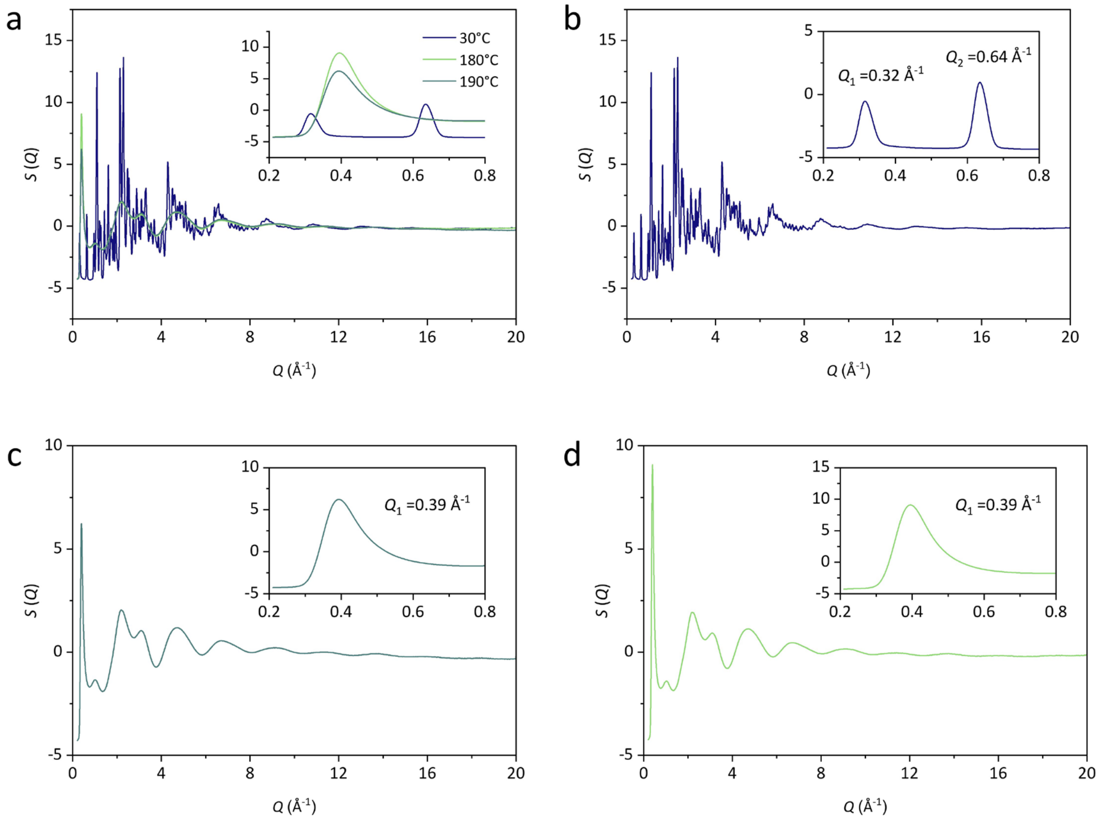

Supplementary Fig. 27. High-temperature X-ray total scattering. (a) Structure factor S(Q) of the crystalline phase (S-NEA)$_2$PbBr$_4$ at 30°C and high-temperature melts at 180°C and 190°C. Individual structure factor S(Q) obtained at (b) 30°C, (c) 180°C and (d) 190°C. Insets show the corresponding low-Q features. Data were collected using the I15-1 beamline at the Diamond Light Source (Oxfordshire, UK) with a wavelength of λ = 0.161669 Å.

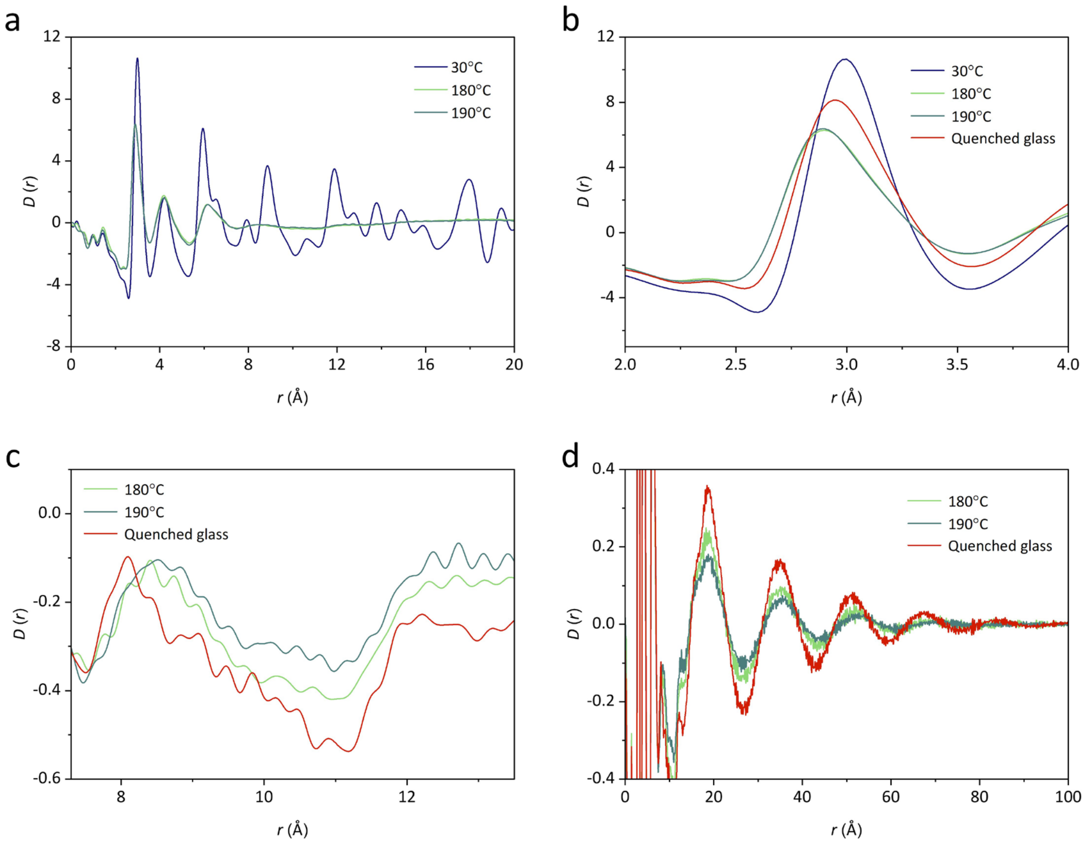

Supplementary Fig. 28. High-temperature X-ray pair distribution functions. (a) Comparison of PDF data of the crystalline (*S*-NEA)$_2$PbBr$_4$ at 30°C and the high-temperature melts at 180°C and 190°C, highlighting (b) the peak at *ca*. 3 Å, (c) the peaks between 8 Å and 13Å, and (d) quasiperiodic oscillations in the extended-*r* range. The PDF of the a_g(*S*-NEA)$_2$PbBr$_4$ glass is also included in comparison with the melts.

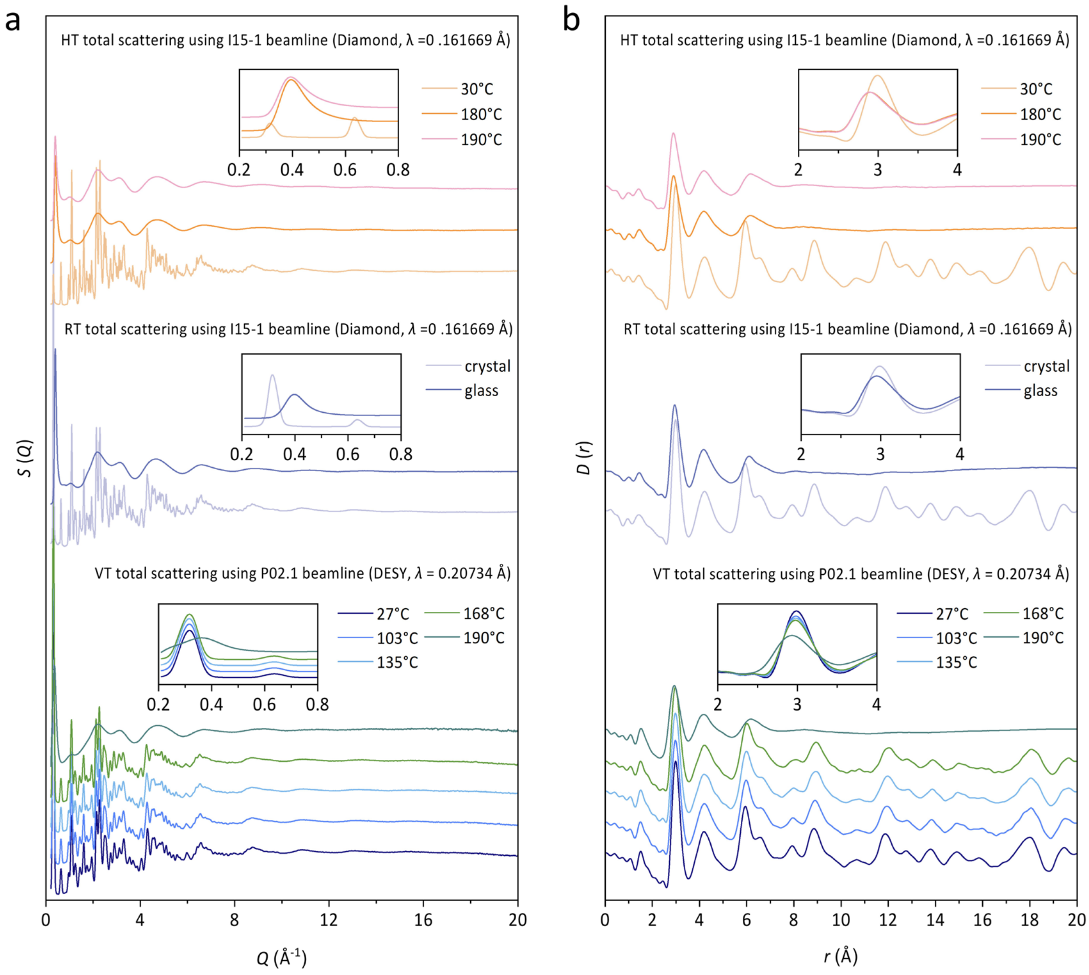

Supplementary Fig. 29. Comparison of X-ray total scattering data of (*S*-NEA)$_2$PbBr$_4$ collected from different beamlines*: (a) Structure factor *S*(*Q*); (b) Pair distribution function *D*(*r*). Room-temperature (RT) total scattering experiments were conducted using the I15-1 beamline at the Diamond Light Source (Oxfordshire, UK) with a wavelength of λ = 0.161669 Å, on both the as-synthesised crystalline sample and the melt-quenched glass sample. High-temperature (HT) total scattering experiments were also performed on the crystalline sample at 180°C and 190°C, both above its melting temperature, to probe the liquid-state structure. To investigate the structural evolution of (*S*-NEA)$_2$PbBr$_4$ upon heating, variable-temperature (VT) total scattering experiments were carried out from 27°C to 190°C using the PETRA III P02.1 beamline at DESY (Hamburg, Germany) with a wavelength of λ = 0.20734 Å.

*The use of two beamlines was necessitated by beamtime availability.

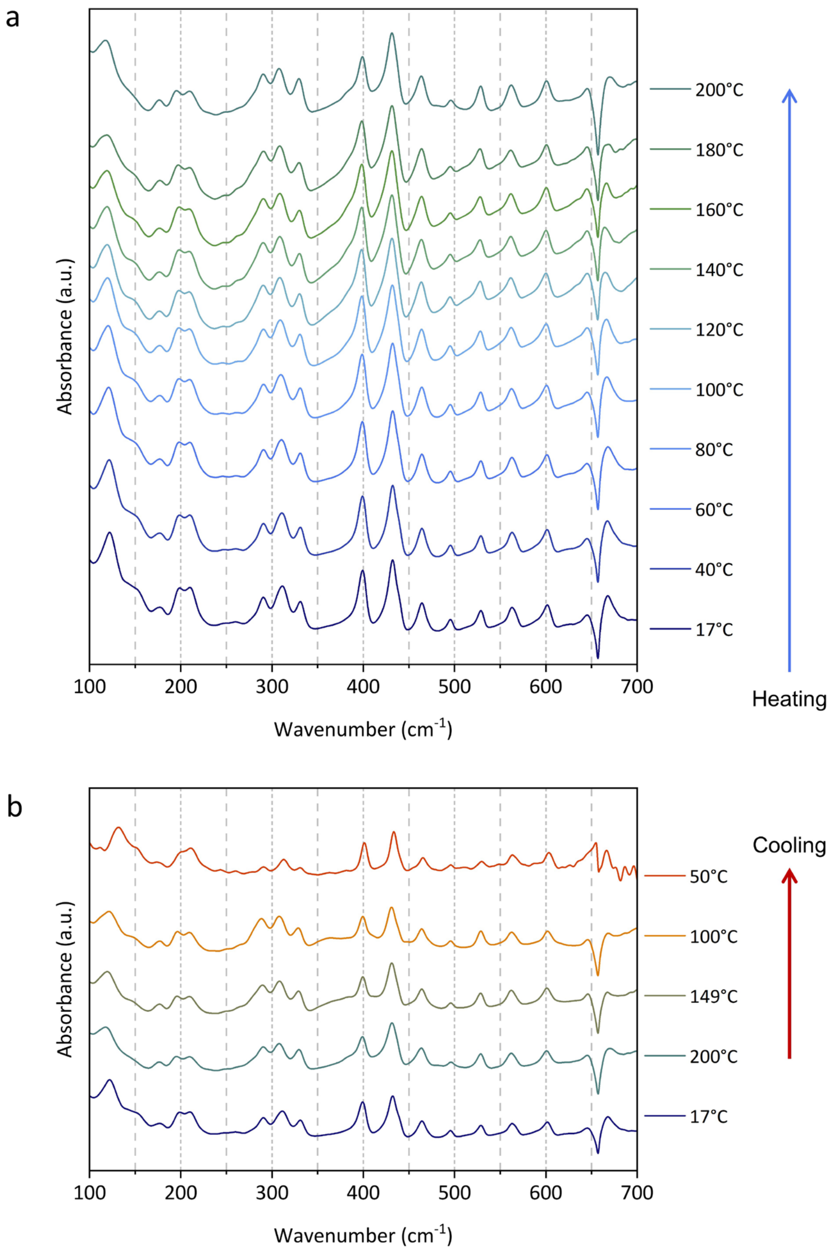

Supplementary Fig. 30. Temperature-resolved *in situ* THz / Far-IR spectra for crystalline (*S*-NEA)$_2$PbBr$_4$ upon (a) heating from 17°C to 300°C and (b) cooling back to 50°C under argon protections. The main features were assigned to Pb-Br stretching within the $[PbBr_6]^{4-}$ at *ca*. 120 cm^{-1} and the aromatic ring deformation between 600-700 cm^{-1}. The absorbance of the 50°C curve in (b) was magnified by 20 times for clarity, and the weak absorbance may arise from the poor scattering of the in situ formed glass piece.

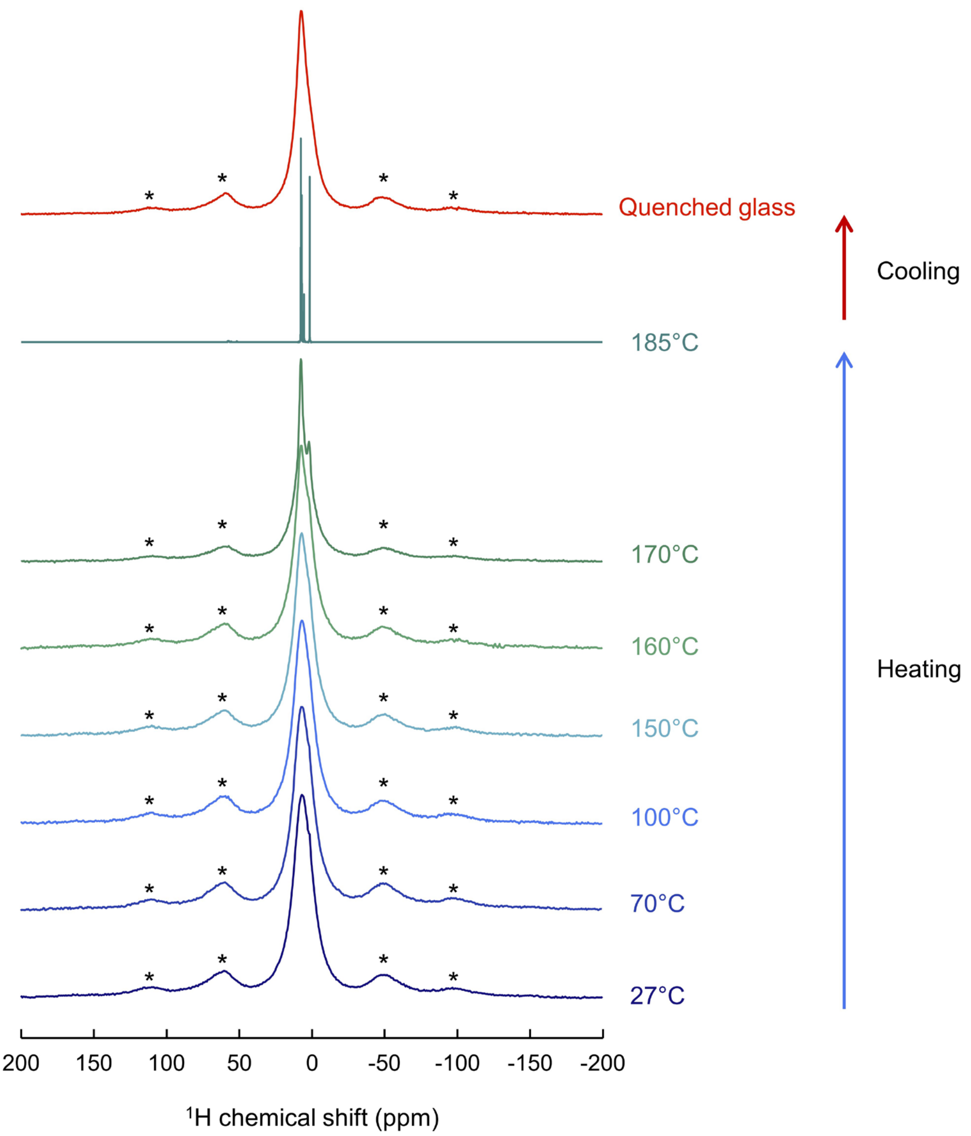

Supplementary Fig. 31. *In situ* variable-temperature ^{1}H MAS NMR spectra for (S-NEA)$_2$PbBr$_4$, recorded upon heating from 27°C to 185°C and subsequent cooling to the quenched glass. All ^{1}H NMR signals are normalised and asterisks (*) denote spinning sidebands.

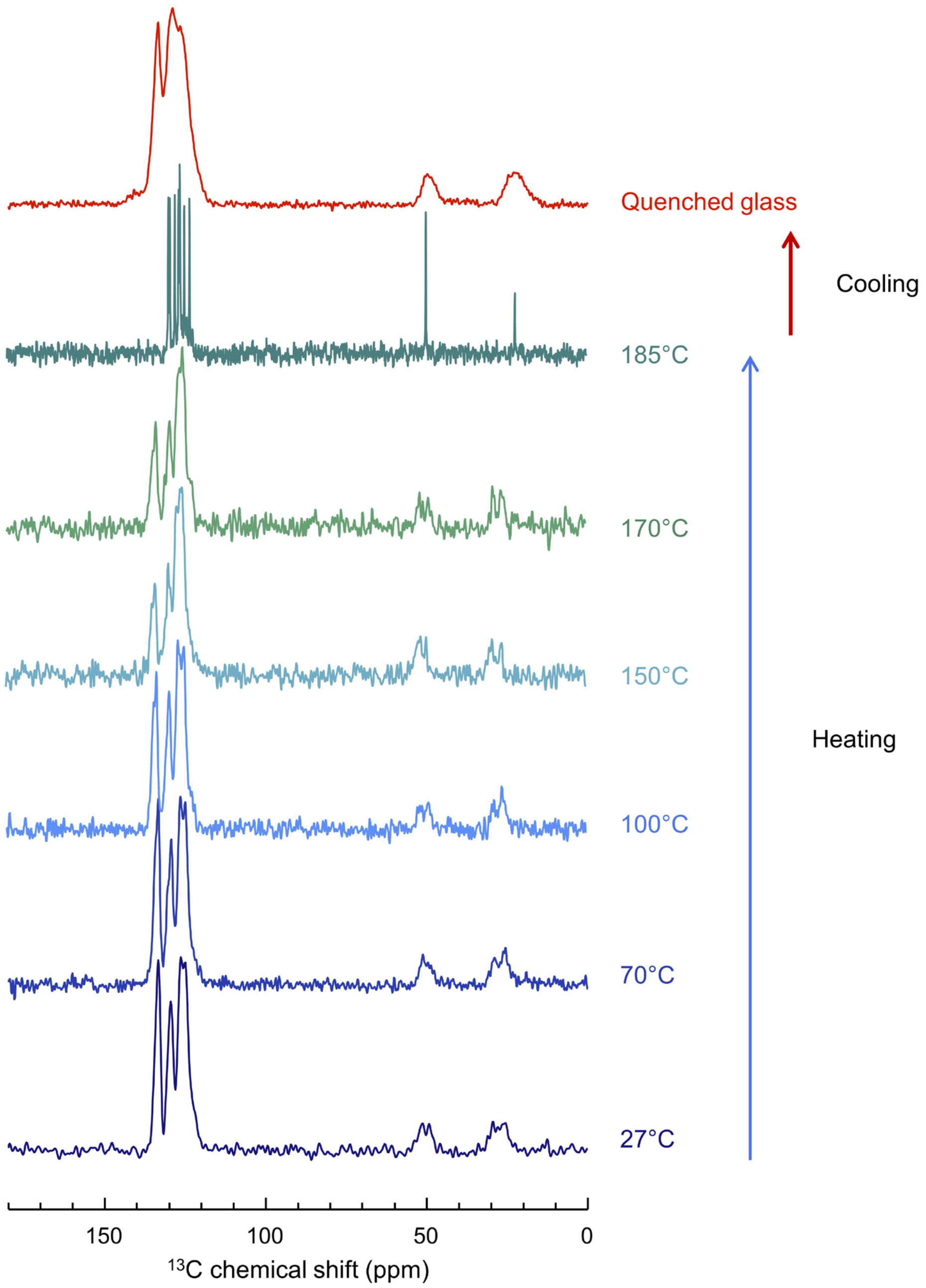

Supplementary Fig. 32. *In situ* variable-temperature ^{13}C MAS NMR spectra for $(S\text{-NEA})_2PbBr_4$, recorded upon heating from 27°C to 185°C and subsequent cooling to the quenched glass. All ^{13}C NMR signals are normalised.

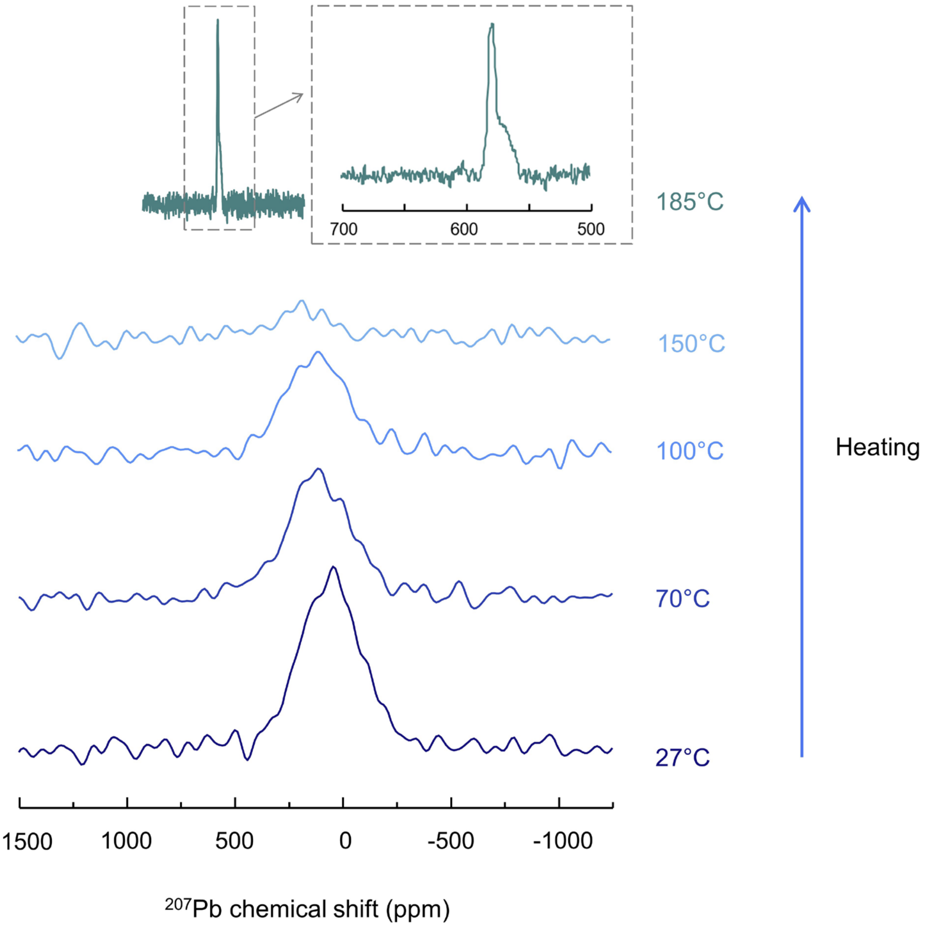

Supplementary Fig. 33. *In situ* variable-temperature ^{207}Pb MAS NMR spectra for (*S*-NEA)$_2$PbBr$_4$, recorded upon heating from 27°C to 185°C. The inset at the top highlights the narrowing of the ^{207}Pb signal at 185°C, suggesting enhanced mobility of Pb nuclei in the melt.

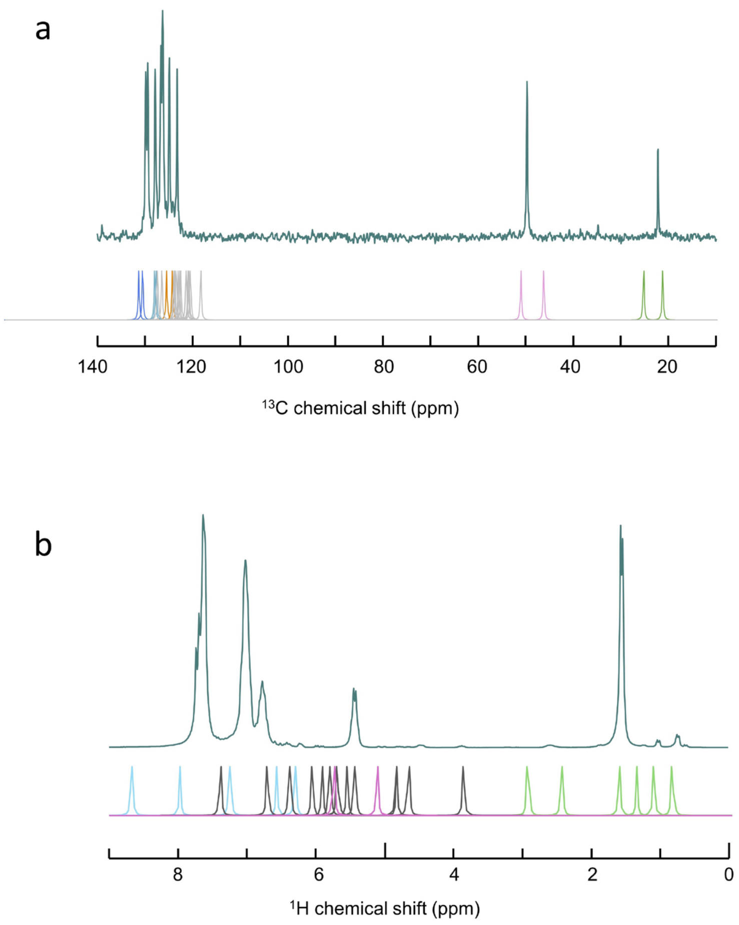

Supplementary Fig. 34. High-temperature (a) ^{13}C and (b) ^{1}H MAS NMR spectra for (*S*-NEA)$_2$PbBr$_4$ liquid at 185°C, shown alongside DFT-calculated chemical shifts for comparison. For details of the DFT assignments, see Supplementary Fig. 20.

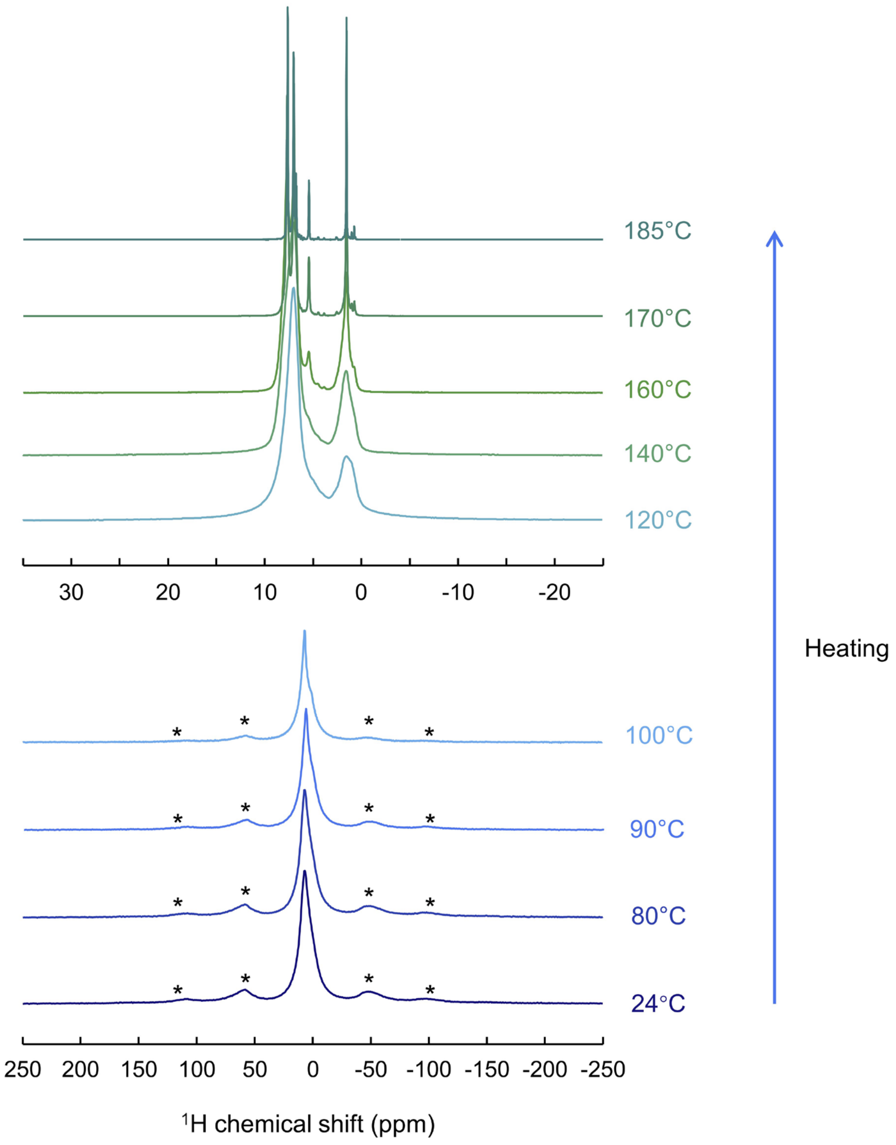

Supplementary Fig. 35. *In situ* variable-temperature ^{1}H MAS NMR spectra for a_g(*S*-NEA)$_2$PbBr$_4$ upon heating from 24°C to 185°C. All ^{1}H NMR signals are normalised and asterisks (*) denote spinning sidebands.

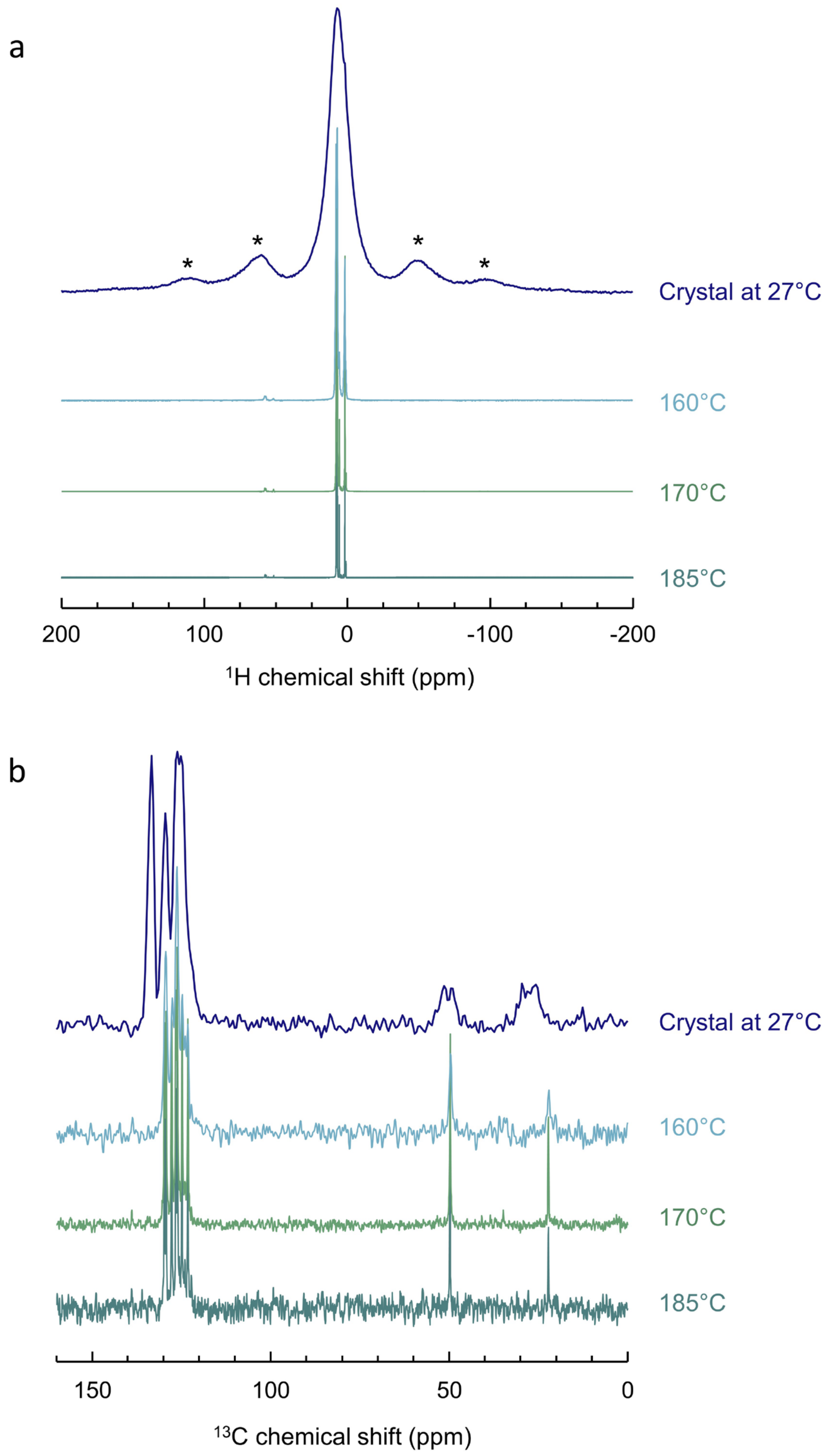

Supplementary Fig. 36. High-temperature (a) ^{1}H and (b) ^{13}C MAS NMR spectra of a_g(*S*-NEA)$_2$PbBr$_4$, recorded upon heating to 160°C, 170°C and 185°C. The corresponding spectra of crystalline sample at 27°C are included for comparison. All ^{1}H and ^{13}C NMR signals are normalised. Asterisks (*) in (a) denote spinning sidebands.

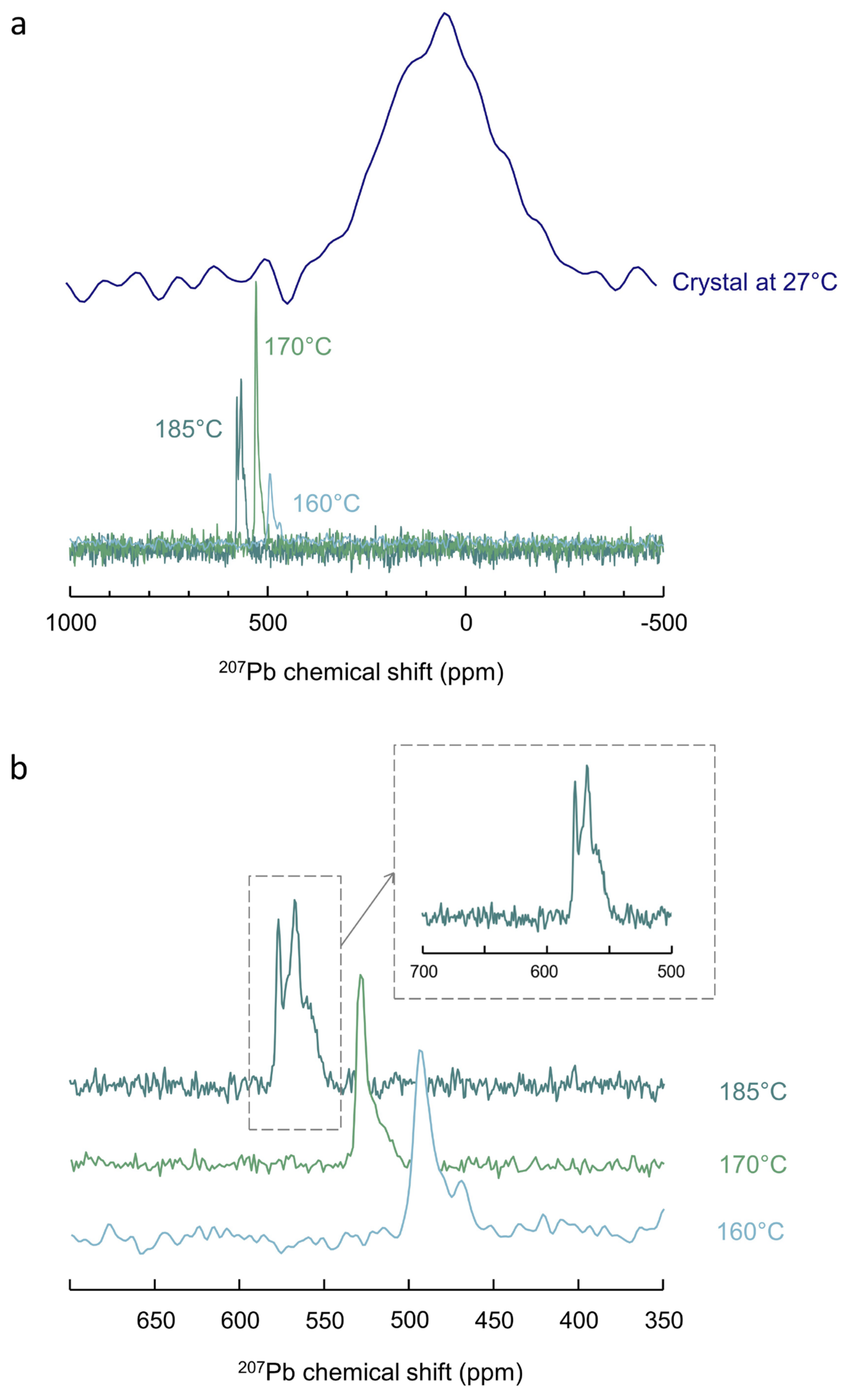

Supplementary Fig. 37. (a) High-temperature ^{207}Pb MAS NMR spectra of a_g(*S*-NEA)$_2$PbBr$_4$, recorded upon heating to 160°C, 170°C and 185°C, with the spectrum of crystalline sample at 27°C shown for comparison. (b) Magnified view of ^{207}Pb MAS NMR spectra at elevated temperatures, with inset highlighting the emergence of multiple signals in the melt at 185°C. In (b), all ^{207}Pb NMR signals are normalised.

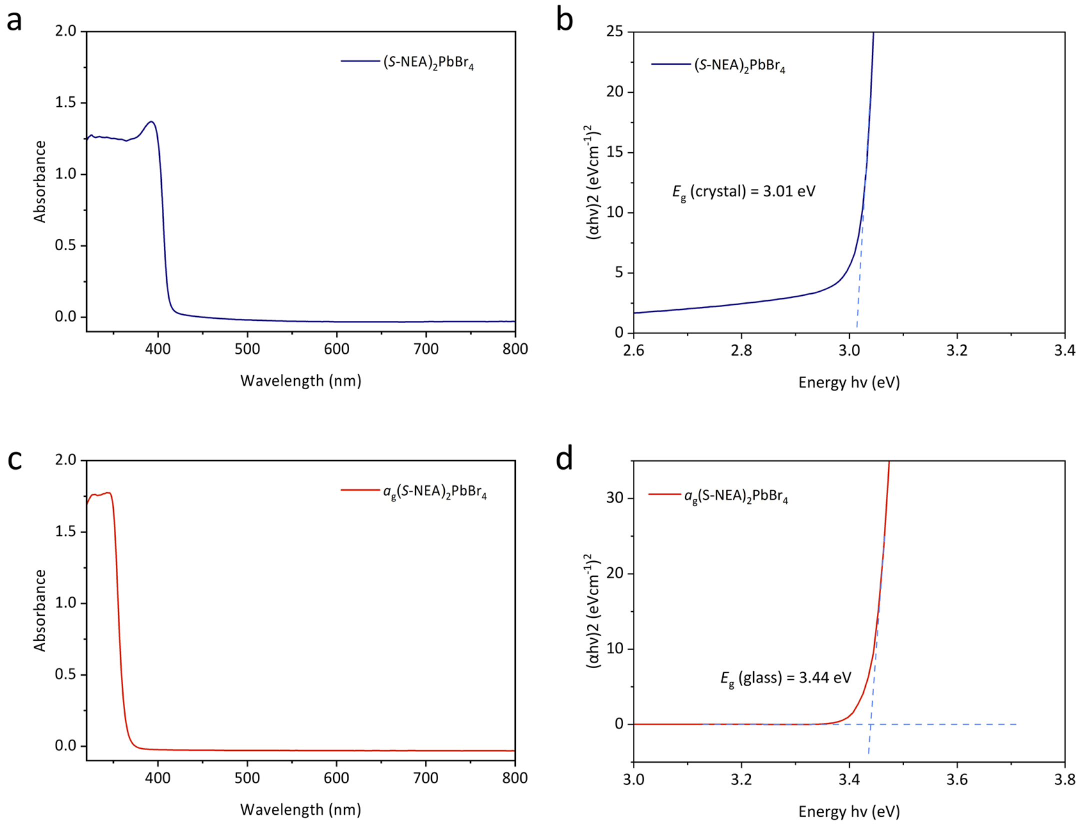

Supplementary Fig. 38. Ambient-temperature UV-vis absorption spectra and corresponding Tauc plots for thin films of (a-b) crystalline (*S*-NEA)$_2$PbBr$_4$ and (c-d) a_g(*S*-NEA)$_2$PbBr$_4$. The optical bandgap (E_g) is determined to be 3.01 eV for the crystalline phase and 3.44 eV for the glassy phase.

Supplementary Table 9. Thickness measurements of samples used in the UV-Vis experiment.

Sample	Thickness (mm)*			
	Position 1	Position 2	Position 3	Average
(*S*-NEA)$_2$PbBr$_4$	0.05	0.04	0.06	0.05
a_g(*S*-NEA)$_2$PbBr$_4$	0.04	0.05	0.07	0.05

* The thickness of each film sample was measured using a micrometer screw gauge with a precision of 0.01 mm, and the average value was calculated based on three measurements at different positions.

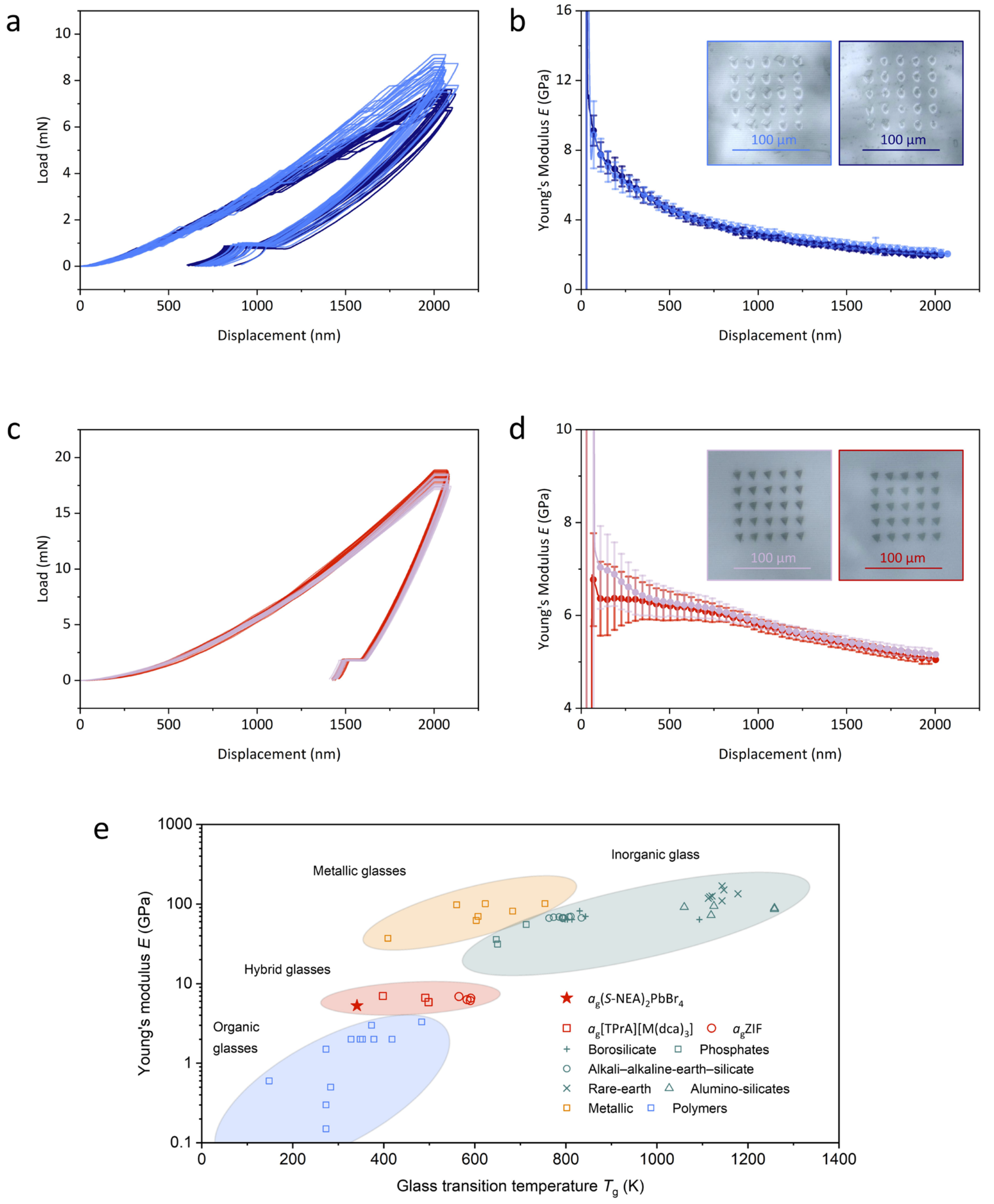

Supplementary Fig. 39. Nanoindentation results for (*S*-NEA)$_2$PbBr$_4$ crystal and glass. (a-b) Load-displacement curves and Young's modulus (*E*) as a function of indentation depth for two crystalline samples. (c-d) Corresponding results for two glassy samples. Insets in (b) and (d) show optical images of the indentation arrays of the respective samples. (e) Comparison of Young's modulus and glass transition temperature (T_g) in various glassy systems, highlighting the positioning of a_g(*S*-NEA)$_2$PbBr$_4$ relative to organic, hybrid, metallic, and inorganic glasses.

Supplementary Table 10

Comparison of the mechanical properties for (S-NEA)$_2$PbBr$_4$ crystal and glass.

Sample	Reduced modulus E_r (GPa)	Young's modulus E (GPa)[b]	Hardness H (GPa)
Crystal[a]	2.39 ± 0.10	2.30 ± 0.10	0.12 ± 0.01
Glass	5.48 ± 0.04	5.29 ± 0.04	0.25 ± 0.01

[a] Measured with the (001) lattice plane facing up.

[b] Assumes Poisson's ratio v = 0.2. Sensitivity analysis shows that increasing v to 0.3 (a 50% increase) results in only a ~ 5% change in E.

References

1. Jana, M. K. et al. Organic-to-inorganic structural chirality transfer in a 2D hybrid perovskite and impact on Rashba-Dresselhaus spin-orbit coupling. *Nat. Commun.* **11**, 4699 (2020).
2. Ye, C. et al. Mechanochemically-induced glass formation from two-dimensional hybrid organic-inorganic perovskites. *Chem. Sci.* **15**, 7198-7205 (2024).
